# Separation-of-Function Alleles of *smc-5* Reveal Domain-Specific Defects and a Conserved Residue Critical for Genome Maintenance

**DOI:** 10.3390/biom15060755

**Published:** 2025-05-23

**Authors:** Haiyan Yuan, Arome Solomon Odiba, Guiyan Liao, Ziteng Zhou, Wenxia Fang, Cheng Jin, Shaojun Li, Xihui Liu, Bin Wang

**Affiliations:** 1 Department of Toxicology, School of Public Health, Guangxi Medical University, Nanning 530021, China; yhy8122@outlook.com; 2 Institute of Biological Sciences and Technology, Guangxi Academy of Sciences, Nanning 530007, Chinazhouziteng158@163.com (Z.Z.); wfang@gxas.cn (W.F.); bwang@gxas.cn (B.W.); 3 Institute of Microbiology, Chinese Academy of Sciences, Beijing 100101, China; 4 Guangxi Key Laboratory of Sugarcane Genetic Improvement, Guangxi Academy of Agricultural Sciences, Nanning 530007, China

**Keywords:** SMC-5/6 complex, *smc-5*, *Caenorhabditis elegans*, genome stability, NSE-1 localization

## Abstract

The SMC-5/6 complex safeguards genome stability through the coordinated action of its core SMC proteins and associated NSE subunits. NSE-1 is a key component of the complex and is essential for DNA repair, yet it remains poorly characterized in *Caenorhabditis elegans*. To further elucidate the functional mechanisms of NSE-1, we performed an EMS-based forward genetic screen in an *nse-1::gfp(wsh1)* reporter strain to identify mutants with defective NSE-1 expression or nuclear localization. We isolated three mutants; *smc-5(wsh31), smc-5(wsh32),* and *smc-5(wsh33)*, that display impaired NSE-1::GFP nuclear localization. SNP mapping and whole-genome sequencing revealed three novel *smc-5* alleles: two truncations, alleles *smc-5(wsh31)* (C587*) and *smc-5(wsh32)* (Q655*), and one missense variant, *smc-5(wsh33)* (Y975D), each altering a highly conserved residue in the SMC domain. All three mutants exhibited significantly reduced brood size, progeny viability, and slightly elevated male percentages. Phenotypic characterization revealed that the truncations completely abrogate NSE-1::GFP nuclear localization, whereas the missense allele causes stage-dependent, partial mislocalization. Functional assays further demonstrated allele-specific and developmental stage-dependent hypersensitivities to DNA-damaging agents (MMS, HU, and cisplatin). These separation-of-function *smc-5* alleles underscore the importance of domains and conserved residues in complex integrity and genome maintenance, and provide powerful genetic tools to dissect SMC-5/6 functions in vivo.

## 1. Introduction

Eukaryotic cells have evolved complex molecular machineries to protect genome integrity, including intricate DNA repair pathways and mechanisms ensuring accurate chromosome segregation during cell division. Central to these processes are the Structural Maintenance of Chromosomes (SMC) protein complexes, which play crucial roles in chromosome organization, condensation, cohesion, and DNA repair [1]. Among these, the SMC-5/6 complex is uniquely implicated in resolving complex DNA structures that arise during DNA replication, recombination, and repair, thereby safeguarding against genomic instability [2]. The SMC-5/6 complex is conserved across eukaryotes and minimally comprises the core SMC-5 and SMC-6 proteins, which heterodimerize via their hinge domains and associate through their ATPase head domains, bridged by the kleisin subunit NSE-4 (Non-SMC Element 4). Additional subunits, including NSE-1, NSE-2 (a SUMO ligase), and NSE-3, associate with this core to form the functional holocomplex [3,4,5]. NSE-1, an essential kleisin-interacting protein with ubiquitin E3 ligase activity potential, is crucial for complex stability and function, often serving as a marker for the complex’s chromosomal localization [6]. Studies in various organisms have demonstrated that dysfunction of the SMC-5/6 complex leads to hypersensitivity to DNA damaging agents, replication stress, defects in homologous recombination repair, chromosome segregation errors, and, in humans, developmental disorders and predisposition to cancer [7,8,9]. Despite its established importance, the precise molecular mechanisms by which SMC-5/6 subunits coordinate their activities, how the complex is recruited to specific chromosomal sites, and how its functions are regulated remain areas of active investigation. In particular, understanding the specific contributions of individual domains and residues within core components like SMC-5 to complex assembly, localization, and functional output requires detailed structure–function analysis. While null mutations demonstrate the essential nature of the complex, they often preclude the study of specific functional aspects that might be retained in separation-of-function or hypomorphic alleles.

The nematode *Caenorhabditis elegans* offers a powerful system to dissect the functions of conserved genome maintenance factors like the SMC-5/6 complex. Our previous work using fluorescently tagged subunits, such as NSE-1::GFP, has visualized the dynamic localization of the complex to chromosomes, particularly in the germline where meiotic recombination and DNA repair are prominent [10,11]. Perturbations leading to mislocalization of tagged subunits often correlate with functional deficits [10,11]. To gain deeper insights into the regulation of SMC-5/6 function and localization, we utilized a *C. elegans* strain expressing NSE-1 tagged with GFP, *nse-1::gfp(wsh1)*, as a reporter for complex integrity and chromosomal association. We performed a forward genetic screen using ethyl methanesulfonate (EMS) mutagenesis to identify mutations causing aberrant NSE-1::GFP distribution in the germline. Here, we report the isolation and characterization of three novel mutant alleles (*smc-5(wsh31)*, *smc-5(wsh32)*, and *smc-5(wsh33)*) exhibiting distinct NSE-1::GFP mislocalization patterns. Through genetic mapping and whole-genome sequencing, we identified causative mutations within the *smc-5* gene, encoding a core component of the complex. These alleles, comprising two truncations (*smc-5(wsh31)* and *smc-5(wsh32)*) and a missense mutation in a highly conserved residue (*smc-5(wsh33)*, Y975D), provide a valuable allelic series. We performed functional analysis, assessing their impact on fertility, progeny viability, chromosome segregation fidelity, and sensitivity to various genotoxic agents (MMS, HU, and Cisplatin) at different developmental stages. Our findings reveal differential requirements for SMC-5 domains and specific residues in maintaining complex integrity, ensuring proper NSE-1 localization, and executing critical DNA repair functions, particularly under conditions of replication stress and interstrand crosslink damage. Our study thus fills a critical knowledge gap by linking a defined SMC-5 domain to the regulation of SMC-5/6 complex assembly and function in vivo.

## 2. Materials and Methods

### 2.1. EMS Mutagenesis of nse-1::gfp(wsh1) Worms

*nse-1::gfp(wsh1)* worms were grown on NGM agar plates seeded with *E. coli* OP50 until most animals reached the L4 stage, at which point they were washed off with M9 buffer into a 15 mL conical tube, briefly centrifuged, and washed once more to remove debris. The final worm pellet was resuspended in 2 mL M9, and in a fume hood a 0.05 M solution of ethyl methanesulfonate (EMS, Sigma #M-0880, Merck KGaA, Darmstadt, Germany) was prepared by adding 10 µL of liquid EMS to 2 mL of M9 in a separate 15 mL tube and gently mixing until the dense liquid dissolved. This EMS solution was combined with the 2 mL worm suspension to yield a final concentration of 0.025 M EMS, sealed with Parafilm, and placed on a rocker at 20 °C for 4 h. All handling and mixing steps were performed using disposable plasticware in a fume hood, and EMS-contaminated items (pipettes, tubes, and residual solutions) were inactivated for 24 h by mixing with an equal volume of 0.1 M NaOH and 20% (*w*/*v*) sodium thiosulfate. After the incubation period, the worms were washed twice with M9, transferred with a Pasteur pipette to the edge of an OP50 lawn (avoiding the use of plastic pipette tips to prevent the worms from sticking), and allowed to recover for 15–20 min, at which point the healthy late-L4 worms were picked as P0 animals. Post-mutagenesis, animals were washed four times in M9 and dispensed onto OP50 plates, where they were grown for two days to lay eggs; the resulting L1 larvae were then filtered as the F1 generation, frozen as a library, and also plated again on OP50 for another two days, after which the next batch of L1 larvae were filtered and designated F2, then similarly frozen as a library.

### 2.2. SNP-Based Mapping Protocol for the Identification of Mutations in the Mutant Strains Isolated

We crossed EMS-induced mutants in the *nse-1::gfp(wsh1)* background to CB4856 (Hawaiian) males and isolated F1 heterozygotes, allowing them to self-fertilize to produce F2 progeny from which homozygous mutant and non-mutant pools were prepared to facilitate “bulk segregant” chromosome mapping, as described by Davis et al. (2005) [12]. For each pool, 30–50 animals were lysed in 20 µL of single-worm lysis buffer (10 mM Tris-HCl pH 8.3, 50 mM KCl, 2.5 mM MgCl_2_, 0.45% NP-40 or IGEPAL CA-630, 0.45% Tween-20, 0.01% gelatin, and 60 µg/mL Proteinase K) by incubation at 65 °C for 1 h followed by 95 °C for 15 min. PCR master mixes were assembled for each SNP marker, with Taq polymerase, dNTPs, and a uniform annealing temperature of 60 °C, and were arrayed into 96-well plates such that mutant and non-mutant lysates could be dispensed into alternate rows. Primers designed to flank DraI-based SNPs at evenly spaced intervals across all six chromosomes were pin-replicated into the master mixes, and after amplification, the PCR products were digested with DraI in the same plate. Samples were analyzed on 2.5% agarose gels, and enrichment of the Bristol allele in the mutant lane was used to infer linkage. Once the candidate chromosome was identified, individual homozygous F2 recombinants were singled and allowed to produce F3 progeny; these populations were each lysed separately, and their templates were again analyzed by PCR and DraI digestion for multiple markers spanning the linked interval. Genotype transitions (from Bristol to Hawaiian or vice versa) among the recombinants pinpointed the region harboring each mutation, enabling fine-scale interval mapping according to the streamlined protocol of Davis et al. [12].

### 2.3. Phenotypic Assessment

To evaluate brood size, 25 L4-stage hermaphrodite worms from each strain were individually placed on NGM plates seeded with OP50 bacteria. Every 12 h, each worm was transferred to a fresh plate, and the number of eggs laid on the previous plate was recorded. This process continued until egg-laying ceased, allowing for the calculation of the total number of fertilized eggs per hermaphrodite. To assess progeny viability, the number of unhatched eggs was counted 24 h post-laying, and viability was expressed as the percentage of hatched larvae relative to the total number of eggs laid. Additionally, 72 h after egg-laying commenced, male progeny were identified and counted to determine the percentage of male offspring.

### 2.4. Genotoxic Assays

To assess the impact of genotoxic agents, synchronized populations of at least 50 *C. elegans* larvae at either the L1 or L4 stage were exposed to varying concentrations of methyl methanesulfonate (MMS), cisplatin, or hydroxyurea (HU), following established protocols [13,14]. For MMS and cisplatin treatments, worms were incubated with the agents at specified doses for 16 h; HU exposure was conducted over a 20 h period. Post-treatment, L1 larvae were allowed a recovery period of 24 h, while L4 larvae recovered for 24 h. For the L1 progeny viability assay, treated larvae were allowed to recover for 72 h (day 3 post-treatment). By this point, individuals capable of maturing had reached the adult stage, whereas developmentally arrested animals were mostly immobile or dead and could be readily excluded. Only the surviving adults were transferred to brood plates for viability scoring. After recovery, five adult worms from each group were individually placed on NGM plates seeded with OP50 bacteria to lay eggs over a 6–8 h period. Parent worms were then removed, and the number of eggs laid was recorded. After an additional 24 h, unhatched eggs were counted to determine progeny viability, calculated as the percentage of hatched larvae relative to the total number of eggs laid. Each experiment was performed in triplicate and repeated independently three times to ensure reproducibility.

### 2.5. Developmental Assays Following Genotoxic Treatment

To assess the impact of DNA-damaging agents on *C. elegans* development, synchronized L1 larvae were obtained by filtering through an 11 μm nylon mesh (Millipore, Burlington, MA, USA) using M9 buffer. These larvae were then exposed to varying concentrations of methyl methanesulfonate (MMS) and cisplatin for 16 h, or hydroxyurea (HU) for 20 h, with specific dosages detailed in the Results section [10]. Post-treatment, the larvae were thoroughly rinsed with M9 buffer to remove residual agents, evenly distributed onto NGM agar plates seeded with OP50 bacteria, and allowed to recover at 20 °C for 48 h. Subsequently, their developmental stages were evaluated under a stereomicroscope. To ensure the reliability and reproducibility of the findings, each experiment was independently conducted twice.

### 2.6. Yeast Two-Hybrid Assay for Interaction Between NSE-4 and SMC-5 (Y975D) Mutant

Cultures of *Y2HGold* yeast cells were first transformed by the lithium acetate method with pairs of plasmids encoding bait (pGBKT7 constructs) and prey (pGADT7 constructs) for NSE-4 or *smc-5* (including the *smc-5* Y975D mutant), using a mixture containing approximately 2.5 µL of each plasmid, 240 µL of 50% PEG3350, 36 µL of 1 M LiAc, and 10 µL of salmon sperm DNA (10 mg/mL). Transformants were spread on synthetic dropout (SD) medium lacking tryptophan and leucine (SD-Trp-Leu) to select for both plasmids, incubated at 30 °C for three days, and single colonies were restreaked to ensure purity. For strain activation and verification, overnight cultures grown in SD-Trp-Leu liquid medium at 30 °C and 200 rpm were adjusted to OD600 ~0.05, then 5 µL of each suspension was spotted onto plates lacking tryptophan and leucine (SD-Trp-Leu) as well as plates additionally lacking histidine (SD-Trp-Leu-His), with or without 3-amino-1,2,4-triazole (3-AT). Growth after 2–3 days at 30 °C was compared to determine whether the bait and prey constructs interacted sufficiently to activate reporter genes, as indicated by colony formation on the more stringent selection media.

### 2.7. Statistical Analysis

To assess the significance of differences within our datasets, we employed statistical tests including the one-way ANOVA with Fisher’s least significant difference (LSD) test, two-way ANOVA with Dunnett’s multiple comparisons test, and two-tailed chi-squared tests with Fisher’s exact test, as appropriate. The specific test applied for each comparison is detailed in the corresponding figure legends. Statistical significance was denoted as follows: *p* > 0.05 (ns) indicating no significant difference, *p* ≤ 0.05 indicating significance at the 5% level, *p* ≤ 0.01 at the 1% level, and *p* ≤ 0.0001 at the 0.01% level. For unpaired two-tailed Student’s *t*-tests, significance levels were set at *p* > 0.05 (ns) for no significant difference, *p* ≤ 0.05 for significance at the 5% level, and *p* ≤ 0.01 at the 1% level. Data are presented as means, with error bars representing the standard error of the mean (SEM).

## 3. Results

### 3.1. EMS Mutagenesis in the nse-1::gfp(wsh1) Strain Uncovers Three Novel Mutations Disrupting NSE-1 Nuclear Distribution in the C. elegans Germline

To investigate the factors influencing the function and distribution of NSE-1, a critical component of the SMC-5/6 complex in *Caenorhabditis elegans*, we performed an EMS mutagenesis screen using a strain carrying the *nse-1::gfp(wsh1)* reporter transgene. From the 26,000 individual F2 progeny screened, we obtained 20 mutants exhibiting altered NSE-1::GFP localization patterns. Interestingly, only three of these mutants were identified as *smc-5* mutants. The remaining mutants, which are not *smc-5*, are currently under active investigation to pinpoint the causative mutations. Each line was outcrossed four times to the parental reporter strain to remove extraneous background mutations. The altered NSE-1::GFP localization pattern persisted after outcrossing, suggesting that each line harbors a single mutation robustly impacting NSE-1 distribution. Under normal conditions, NSE-1::GFP localizes predominantly to chromosomes within the nuclei of germline cells, consistent with its established role within the SMC-5/6 complex, which is essential for chromosome organization, DNA repair, and genome stability [1,2,3]. In control gonads, germline nuclei exhibit a uniform GFP signal associated with chromatin through pachytene and subsequent stages of gametogenesis, often displaying clear, bright foci demarcating chromosomal structures.

**Figure 1:**
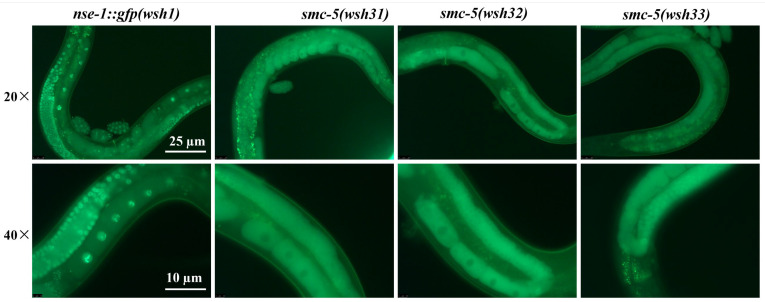
Mutations *smc-5(wsh31)*, *smc-5(wsh32)*, and *smc-5(wsh33)* cause aberrant NSE-1::GFP localization in the *C. elegans* germline. Fluorescence microscopy images comparing germline nuclei from the parental *nse-1::gfp(wsh1)* control strain and the indicated mutants (*smc-5(wsh31)*, *smc-5(wsh32)*, and *smc-5(wsh33)*). Representative images acquired with 20× (upper row) and 40× (lower row) objectives are shown for each genotype. In the parental control strain, NSE-1::GFP exhibits clear localization to chromosomes within the nuclei. In contrast, NSE-1::GFP signal is largely excluded from the nucleus in the *smc-5(wsh31)* and *smc-5(wsh32)* mutants. The *smc-5(wsh33)* mutant displays partial mislocalization, characterized by reduced chromosomal association compared to the control. Images were captured using a Leica DM6B fluorescence microscope. “wsh” is the allele designation assigned by the Caenorhabditis Genetics Center (CGC) to our lab.

By contrast, each of the three EMS-derived mutant lines displayed a distinct mislocalization phenotype (Figure 1). The *smc-5(wsh31)* and *smc-5(wsh32)* mutants exhibited similar phenotypes, characterized by a lack of discernible NSE-1::GFP signal within the nucleus. This phenotype is reminiscent of prior observations where perturbation of SMC-5/6 subunits led to partial cytoplasmic accumulation of complex components [10,15]. We hypothesized that *smc-5(wsh31)* and *smc-5(wsh32)* might carry lesions in a gene required for the stable loading or retention of NSE-1 onto chromatin; loss of such a factor could impair the normal recruitment of NSE-1 to meiotic chromosomes. Alternatively, the underlying mutation could destabilize the NSE-1::GFP fusion protein itself, reducing its effective concentration within the nucleus. Meanwhile, in *smc-5(wsh33)* mutants, NSE-1::GFP exhibited a partial redistribution away from chromosomes during most germline stages. This suggested that the *smc-5(wsh33)* mutation might affect essential factors involved in SMC-5/6 complex assembly, stability on chromatin, or nucleocytoplasmic trafficking. Furthermore, the overall NSE-1::GFP fluorescence intensity appeared reduced in all three mutants compared to the parental strain. This observation could potentially indicate decreased protein expression, reduced protein stability, or an altered subcellular distribution resulting in signal dilution within the focal plane.

### 3.2. Chromosome and Interval Mapping of Mutations Affecting NSE-1::GFP Distribution in smc-5(wsh31), smc-5(wsh32), and smc-5(wsh33) Mutants

**Figure 2:**
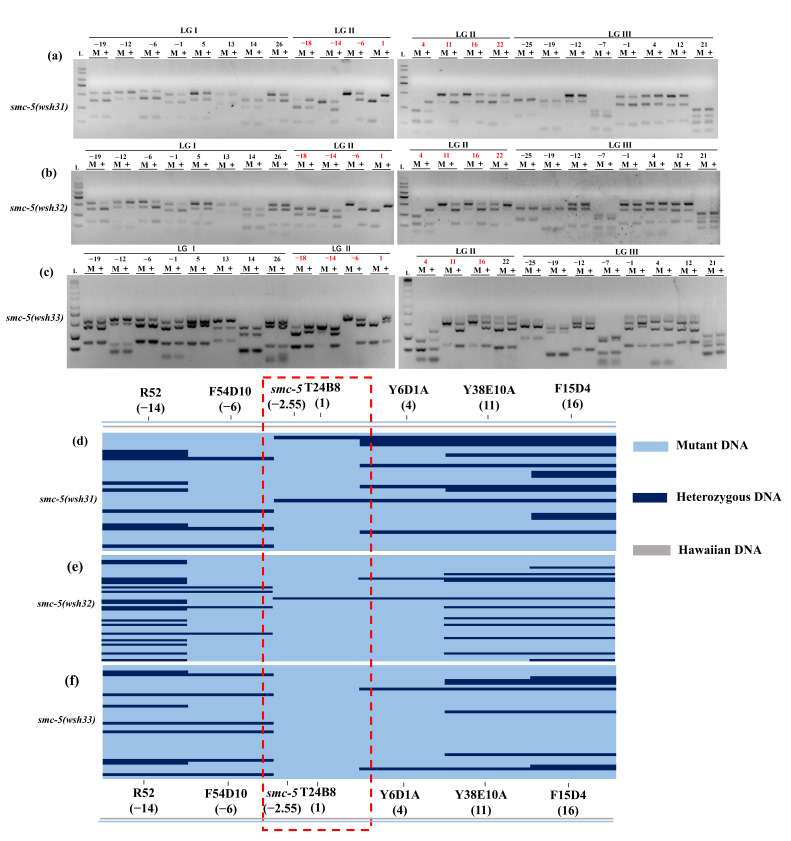
Chromosome and interval mapping of mutations in *smc-5(wsh31)*, *smc-5(wsh32)*, and *smc-5(wsh33)*. (**a**–**c**) Chromosome mapping results for *smc-5(wsh31)* (**a**), *smc-5(wsh32)* (**b**), and *smc-5(wsh33)* (**c**). Agarose gels display DraI-digested PCR products for 48 SNPs across chromosomes I, II, III, IV, V, and X, with genetic positions shown in red (-18, -14, -4, 1, 4, 11, 16, 22) (Figures S1–S3). Lanes are labeled “M” (mutant) and “+” (wild-type). On chromosome II, mutant lanes exhibit enrichment of Bristol-specific bands, indicating linkage, whereas other chromosomes show identical patterns between mutant and wild-type lanes. (**d**–**f**) Interval mapping results for *smc-5(wsh31)* (**d**), *smc-5(wsh32)* (**e**), and *smc-5(wsh33)* (**f**) on chromosome II. Each row represents an individual recombinant, and each column corresponds to a chromosome II SNP. Blue indicates homozygous Bristol (N2), purple indicates heterozygous (N2/CB4856), and grey indicates homozygous Hawaiian (CB4856). The red dashed rectangle delineates the region of minimal recombination, indicative of tight linkage to the mutations.

To identify the genetic loci responsible for the altered NSE-1::GFP distribution observed in the *smc-5(wsh31)*, *smc-5(wsh32)*, and *smc-5(wsh33)* mutants, we employed a two-step single nucleotide polymorphism (SNP) mapping strategy using the Hawaiian strain CB4856, following the methodology described by Davis et al. [12]. The agarose gel results for chromosomes I, II, and III mapping are shown in Figure 2a–c for *smc-5(wsh31)*, *smc-5(wsh32)*, and *smc-5(wsh33)*, respectively. The results indicate that the mutations map to chromosome II, as evidenced by the genetic positions highlighted in red: *smc-5(wsh31)* (-18, -14, -4, 1, 4, 11, 16, 22), *smc-5(wsh32)* (-14, -4, 1, 4, 11, 16, 22), and *smc-5(wsh33)* (-18, -14, -4, 1, 4, 11, 16). Differences in banding patterns between mutant and wild-type lanes were observed across the respective genetic positions indicated on chromosome II, suggesting strong linkage to this chromosome. Conversely, for chromosomes I, III, IV, V, and X, the banding patterns were identical between the mutant and wild-type samples, indicating no linkage to these chromosomes (Figures S1–S3). PCR lanes are labeled “M” for mutant and “+” for wild-type. On chromosome II, a clear difference in banding patterns between mutant and wild-type lanes was observed across all genetic positions, reinforcing strong linkage to chromosome II. These results suggest that the mutations affecting NSE-1::GFP distribution in *smc-5(wsh31)*, *smc-5(wsh32)*, and *smc-5(wsh33)* are located on chromosome II, potentially affecting the same or closely related genetic loci.

To refine the location of these mutations, interval mapping was performed on chromosome II for each mutant strain (Figure 2d–f). Ninety-six F2 mutant animals from the heterozygous F1 progeny of each cross were isolated, allowed to self-fertilize, and their F3 progeny lysed in a 96-well plate. DNA templates were pin-replicated into PCR plates containing primers for six chromosome II SNPs: R52, F54D10, T24B8, Y6D1A, Y38E10A, and F15D4, corresponding to genetic positions -14, -6, 1, 4, 11, and 16, respectively. PCR amplification, DraI digestion, and gel electrophoresis were conducted to determine the genotype of each individual recombinant and identify recombination breakpoints. Interval mapping results for *smc-5(wsh31)*, *smc-5(wsh32)*, and *smc-5(wsh33)* are shown in Figure 2d–f, respectively. Each panel displays the genotypes of individual recombinants across six chromosome II SNPs. A vertical (red dashed) rectangular outline highlights the region of minimal recombination, indicative of tight linkage to the mutation. The majority of recombinants were homozygous Bristol across all SNPs, reflecting the strong linkage observed in the chromosome mapping. A subset of recombinants displayed recombination events, with the region between genetic positions -6 (F54D10) and 4 (Y6D1A), particularly closer to 1 (T24B8), exhibiting the least recombination. Most recombinants remained homozygous Bristol within this interval, while recombination events (indicated by heterozygous or Hawaiian genotypes) were more frequent outside this region. This pattern indicates that the mutation lies between -4 and 1 on chromosome II. The consistency of this mapping interval across all three mutants strongly suggests that the mutations in *smc-5(wsh31)*, *smc-5(wsh32)*, and *smc-5(wsh33)* are located within the same genetic region on chromosome II, potentially affecting the same gene or closely linked genes. Examination of the genetic map of chromosome II identified the *smc-5* gene as a potential candidate within the -4 to 1 interval. The *smc-5* gene is located at approximately -2.5 on chromosome II, which falls within the mapped interval between -6 (F54D10) and 4 (Y6D1A), and closest to 1 (T24B8).

### 3.3. Whole-Genome Sequencing Identifies Mutations in smc-5 in smc-5(wsh31), smc-5(wsh32), and smc-5(wsh33) Mutants

**Figure 3:**
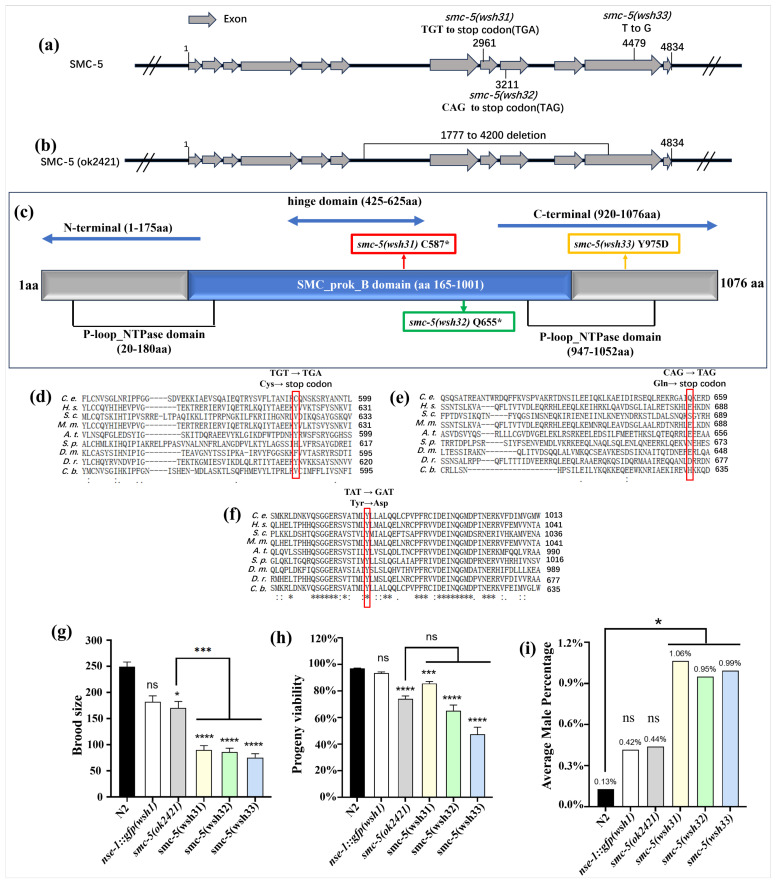
Identification and characterization of *smc-5* mutations in *smc-5(wsh31)*, *smc-5(wsh32)*, and *smc-5(wsh33)*. (**a**) Schematic of the *smc-5* gene structure on chromosome II, showing the positions of the mutations in *smc-5(wsh31)* (C587*), *smc-5(wsh32)* (Q655*), and *smc-5(wsh33)* (Y975D). Exons are depicted as boxes, with the SMC domain highlighted in grey. (**b**) Comparison with the *smc-5(ok2421)* deletion allele, which removes a large portion of the SMC domain. (**c**) Mapping of the mutations onto the SMC-5 protein, showing their positions within the SMC domain (Figure S4b). (**d**–**f**) Amino acid sequence alignments of SMC-5 regions across *C. elegans*, *H. sapiens*, *S. cerevisiae (yeast)*, *M. musculus*, *A. thaliana*, *S. pombe*, *D. melanogaster*, *D. rerio*, and *C. briggsae*. Red vertical rectangular boxes highlight the position of the amino acid residue affected. (**g**) Brood size of N2, *nse-1::gfp(wsh1)*, *smc-5(ok2421)*, *smc-5(wsh31)*, *smc-5(wsh32)*, and *smc-5(wsh33)* mutants, showing significant reductions in all mutants compared to N2. (**h**) Progeny viability, measured as the percentage of viable offspring, with all mutants showing reduced viability compared to N2. (**i**) Male frequency, indicating increased X-chromosome nondisjunction in all the new mutants compared to N2, *nse-1::gfp(wsh1)*, and *smc-5(ok2421)*. Data are presented as mean ± SEM; statistical significance was determined by Student’s *t*-test (* *p* < 0.05, *** *p* < 0.001, **** *p* < 0.0001, ns: not significant). Number of animals *n*; N2 = 28, *nse-1::gfp(wsh1)* = 25, *smc-5(ok2421)* = 28, *smc-5(wsh31)* = 25, *smc-5(wsh32)* = 22, *smc-5(wsh33)* = 24 (Table S1). “wsh” is the allele designation assigned by the CGC to our lab.

Total genomic DNA was extracted from the three mutants, followed by whole-genome sequencing (WGS) and bioinformatics analysis. The sequencing revealed distinct single nucleotide mutations in *smc-5* for each mutant, designated as *smc-5(wsh31)*, *smc-5(wsh32)*, and *smc-5(wsh33)*. The *smc-5(wsh31)* mutation results in a C587* nonsense mutation that introduces a premature stop codon, truncating the protein at amino acid 587 within the SMC domain (Figure 3a). The *smc-5(wsh32)* allele carries a Q655* nonsense mutation, causing truncation at amino acid 655 in the SMC-5 domain, likely disrupting its incorporation into the SMC-5/6 complex. Lastly, *smc-5(wsh33)* harbors a Y975D missense mutation, replacing a tyrosine with an aspartic acid at position 975 within the SMC domain. This substitution may affect the structural conformation or interaction properties of SMC-5, impairing its function within the complex. To better understand the impact of these mutations, their positions were mapped relative to the functional domains of the SMC-5 protein (Figure 3c and Figure S4). The protein contains a conserved SMC domain that includes N-terminal and C-terminal globular regions connected by a coiled-coil segment required for dimerization with SMC-6 and DNA binding [16,17]. The truncations caused by *smc-5(wsh31)* (C587) and *smc-5(wsh32)* (Q655) occur within the SMC domain, likely producing non-functional proteins unable to form complexes. In contrast, the *smc-5(wsh33)* (Y975D) mutation near the C-terminal SMC domain may disrupt critical interactions or structural stability, impairing its functional integrity [18,19].

Studies have demonstrated that mutations within the SMC domain of SMC-5/6 complex components can destabilize the entire complex, leading to mislocalization of NSE-1 and other subunits [15]. The identification of these new *smc-5* alleles (*smc-5(wsh31)*, *smc-5(wsh32)*, and *smc-5(wsh33)*) represents a significant advancement in the study of SMC-5/6 complex function. While null alleles like *smc-5(ok2421)* have provided insights into the complete loss of SMC-5 function, they do not allow detailed dissection of specific functional domains or residues. These new alleles offer opportunities for structure–function analyses of SMC-5, providing insights into molecular mechanisms through experimental analyses such as genotoxic stress assays. To contextualize these mutations, a comparison was drawn with the *smc-5(ok2421)* deletion allele (Figure 3b,c). Unlike *smc-5(ok2421)*, which causes a null phenotype, the new mutations enable nuanced exploration of SMC-5 domains and residues. For instance, the *smc-5(wsh33)* (Y975D) missense mutation offers a unique opportunity to examine the role of tyrosine at position 975 in SMC-5 function. Truncations caused by *smc-5(wsh31)* and *smc-5(wsh32)* may help define the minimal domains required for SMC-5/6 complex assembly and function.

The evolutionary importance of the mutated residues was assessed through amino acid sequence alignments of SMC-5 across multiple species, including *Caenorhabditis elegans*, *Homo sapiens* (human), *Saccharomyces cerevisiae* (yeast), *Mus musculus* (mouse), *Arabidopsis thaliana* (plant), *Schizosaccharomyces pombe* (yeast), *Drosophila melanogaster* (fruit fly), *Danio rerio*, and *Caenorhabditis briggsae* (Figure 3d–f). The Y975 residue is fully conserved, with tyrosine consistently present across all species examined. Tyrosine residues are often involved in protein–protein interactions, phosphorylation, or hydrophobic stabilization of protein structures. The strict conservation of Y975 underscores its critical role in SMC-5 function, likely mediating interactions within the SMC-5/6 complex or with DNA. The Y975D mutation replaces a neutral, aromatic tyrosine with a negatively charged aspartic acid, potentially disrupting these interactions. Conserved residues are typically under strong selective pressure due to their essential roles in protein function, stability, and interactions [20,21]. Mutations at such sites often have profound phenotypic consequences, as they interfere with evolutionarily optimized features of the protein [20,21]. The phenotypes observed in *smc-5(wsh33)* mutants highlight the importance of these residues, as their disruption leads to defects in NSE-1 localization, reflecting the diverse roles of SMC-5 in complex assembly, DNA binding, and chromosome organization. Understanding these conserved residues also has implications for studying SMC-5/6 function in higher organisms, including humans. Mutations in human *SMC5* have been associated with chromosomal instability and cancer. Investigating conserved residues such as Y975 may provide insights into disease mechanisms and inform therapeutic strategies.

### 3.4. Functional Analysis of smc-5 Alleles Reveals Defects in Brood Size, Progeny Viability, and Male Frequency

To investigate the functional consequences of the newly identified *smc-5(wsh31)*, *smc-5(wsh32)*, and *smc-5(wsh33)* alleles, three key phenotypic readouts were quantified: brood size, progeny viability, and spontaneous male frequency. Comparisons were made with wild-type (N2), the *nse-1::gfp(wsh1)* control (phenotypically equivalent to N2 in these assays), and the *smc-5(ok2421)* null allele. In contrast to the robust brood size observed in N2 (*p* ≥ 0.05 vs. *nse-1::gfp(wsh1)*), the *smc-5(ok2421)* mutants showed a significant reduction (*p* < 0.001) (Figure 3g). The three new *smc-5* alleles, *smc-5(wsh31)*, *smc-5(wsh32)*, and *smc-5(wsh33)*, also produced markedly fewer progeny than N2 (*p* < 0.001). Furthermore, the brood size of each new allele was significantly lower than that of *smc-5(ok2421)*, suggesting that these mutations disrupt the *smc-5*-mediated functions essential for normal gametogenesis. Consistent with the observed brood size defects, *smc-5(ok2421)* mutants exhibited significantly reduced embryonic and larval survival (*p* < 0.001 vs. N2) (Figure 3h). Each of the newly identified alleles also displayed reduced viability relative to N2; however, the severity of the viability defects did not differ significantly from that of *smc-5(ok2421)*.

To further assess the fidelity of chromosome segregation, the frequency of male progeny was examined. An increased male percentage in *C. elegans* is indicative of X-chromosome nondisjunction during meiosis, a hallmark of defective chromosome segregation. Under standard hermaphrodite self-fertilization conditions, spontaneous male production in N2 is minimal (~0.1–0.2%), reflecting low rates of spontaneous nondisjunction. In contrast, the new mutants exhibited slightly elevated male frequencies, with *smc-5(wsh31)*, *smc-5(wsh32)*, and *smc-5(wsh33)* each producing ~1.0% male progeny (Figure 3i). These findings indicate that all three alleles are compromised in their ability to maintain faithful meiotic or mitotic chromosome dynamics, consistent with the established role of the SMC-5/6 complex in genome stability [15,15]. In summary, the newly identified *smc-5* alleles exhibit severe defects in fertility, progeny viability, and chromosome segregation fidelity, underscoring the critical role of *smc-5* in these essential biological processes.

### 3.5. Allele-Specific Sensitivities of smc-5(wsh31), smc-5(wsh32), and smc-5(wsh33) to MMS, HU, and Cisplatin Reveal Partial vs. Null-Like Phenotypes in C. elegans

**Figure 4:**
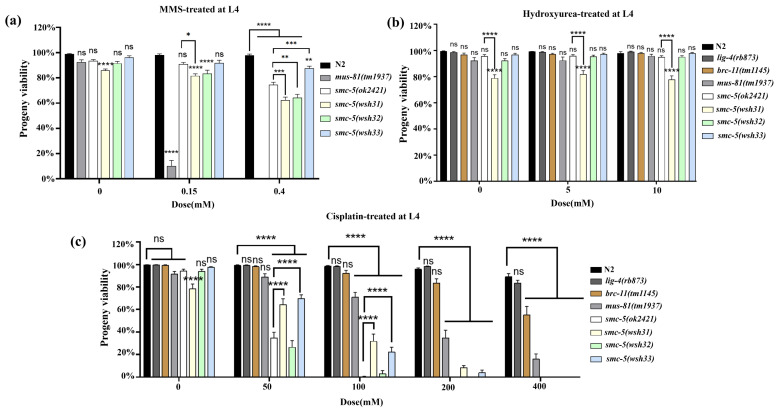
Differential sensitivities of *smc-5* alleles to genotoxic agents. (**a**) Viability assays of L4-stage *C. elegans* exposed to varying concentrations of methyl methanesulfonate (MMS). (**b**) Viability assays of L4-stage worms subjected to replication stress induced by hydroxyurea (HU). (**c**) Viability assays of L4-stage worms exposed to escalating concentrations of cisplatin, which induces inter- and intra-strand DNA crosslinks. Bars represent the mean ± SEM for each genotype, based on three independent biological replicates. Statistical significance was evaluated using one-way ANOVA with multiple-comparison corrections. Asterisks indicate levels of significance relative to N2 or the appropriate control group, as detailed in the text (* *p* < 0.05, ** *p* < 0.01, *** *p* < 0.001, **** *p* < 0.0001, ns: not significant). Sample sizes (n) are available in Tables S2–S4 for (**a**–**c**), respectively. No significant difference was observed between N2 and *nse-1::gfp(wsh1)*, indicating that the GFP tag did not affect the assay outcome (Figure S5).

Given the established roles of the SMC-5/6 complex in DNA repair and genome stability, we sought to determine the response of the newly identified *smc-5(wsh31)*, *smc-5(wsh32)*, and *smc-5(wsh33)* alleles to genotoxic stress. Alkylating agents such as methyl methanesulfonate (MMS) induce DNA lesions that require the SMC-5/6 complex for efficient repair. In addition to N2 and *smc-5(ok2421)*, the DNA repair mutant *mus-81(mn193)*, known for high MMS sensitivity was included. Exposure to 0.15 mM MMS showed N2 retaining high viability (>95%), while *mus-81(mn193)* exhibited a dramatic collapse in viability (*p* < 0.0001 vs. N2) (Figure 4a). Among the new *smc-5* alleles, *smc-5(wsh31)* and *smc-5(wsh32)* displayed intermediate sensitivity, whereas *smc-5(wsh33)* remained resilient. Increasing the MMS dose to 0.4 mM amplified these trends. N2 showed continued resistance (~90–100% viability), while *mus-81(mn193)* dropped to zero percent viability. The *smc-5(wsh31)* and *smc-5(wsh32)* mutants approached null-like sensitivity at higher MMS doses, whereas *smc-5(wsh33)* maintained greater resilience. These graded responses suggest that the missense mutation in *smc-5(wsh33)* retains partial functionality in MMS-induced DNA damage repair, while the two truncation mutations impair complex integrity more severely. This observation aligns with earlier structure–function analyses that revealed that complete or partial domain loss disrupts SMC-5 activity [22].

To further evaluate the DNA repair functionality of the new *smc-5* alleles under replication stress, L4-stage worms were exposed to hydroxyurea (HU). Hydroxyurea depletes deoxyribonucleotide pools and stalls replication forks, eliciting a replication-stress response in which the SMC-5/6 complex plays a critical stabilizing role. None of the strains displayed HU sensitivity at the L4 stage treatment, and even *smc-5(wsh31)*, which showed some differences compared to the control, did not vary significantly between doses (*p* > 0.05) (Figure 4b). This result is consistent with prior studies showing that L4 pachytene cells are not undergoing DNA replication, limiting the effects of deoxyribonucleotide depletion. A more accurate assessment of HU sensitivity would likely require L1 assays, in which the replication stress response is more pronounced. We next examined the response of each strain to cisplatin-induced DNA lesions. Cisplatin primarily forms inter- and intra-strand crosslinks that challenge replication fork progression and require multiple repair pathways, including those mediated by the SMC-5/6 complex. Alongside wild-type N2, we tested *lig-4(rb873)* (a mutant key to non-homologous end joining), *brc-11(tm1145)* (homologous to human BRCA2), *mus-81(mn193)* (a structure-specific endonuclease mutant), and the null *smc-5(ok2421)* control, which served as the benchmark for differentiating between partial- and complete-loss-of-function phenotypes. Cisplatin-treated groups at concentrations of 50 mM and 100 mM (Figure 4c) revealed a dose-dependent viability drop across all SMC-5-defective backgrounds, with *smc-5(ok2421)* exhibiting the most dramatic sensitivity (*p* < 0.0001 vs. N2) at 100 mM. At 200 mM cisplatin, viability in SMC-5-deficient strains dropped sharply. N2 maintained measurable viability (80–90%), while *smc-5(ok2421)* and *smc-5(wsh32)* approached complete lethality (*p* < 0.0001 vs. N2). At the same dose, *smc-5(wsh31)* and *smc-5(wsh33)* also exhibited near-complete lethality. At 400 mM cisplatin, all *smc-5* mutants showed zero percent viability. For all these assays, a comparison between N2 and *nse-1::gfp(wsh1)* revealed no significant difference, indicating that the GFP tag did not influence the assay outcome (Figure S5). Taken together, the MMS, HU, and cisplatin assays provide valuable insights into allele-specific differences in SMC-5/6 function. These data suggest that the new *smc-5* alleles offer a unique opportunity to dissect the structure–function relationships within this essential DNA repair complex.

### 3.6. L1-Stage DNA Damage Assays Uncover Differential Replication Stress Sensitivities of Novel smc-5 Alleles

**Figure 5:**
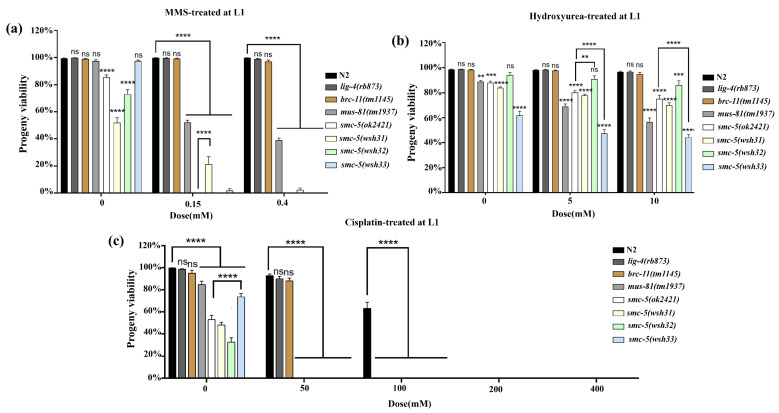
DNA damage sensitivity of L1-stage wild-type and DNA repair mutant *C. elegans* strains, measured by progeny viability. Percent progeny viability following exposure to varying doses of (**a**) methyl methanesulfonate (MMS; alkylating agent), (**b**) hydroxyurea (HU; replication stress), and (**c**) cisplatin (inter- and intra-strand crosslinks). Asterisks indicate levels of significance relative to N2 or the appropriate control group, as detailed in the text (** *p* < 0.01, *** *p* < 0.001, **** *p* < 0.0001, ns: not significant). Sample sizes (n) are available in Tables S5–S7 for (**a**–**c**), respectively. No significant difference was observed between N2 and *nse-1::gfp(wsh1)*, indicating that the GFP tag did not affect the assay outcome (Figure S6).

We performed progeny viability assays on animals exposed at the L1 larval stage, a developmental period marked by rapid cell proliferation in somatic and germline tissues. L1 larvae provide a unique opportunity to probe replication-associated DNA repair defects, complementing L4-stage exposure assays by uncovering vulnerabilities during early development that may remain masked at later stages. Methyl methanesulfonate (MMS) treatment at the L1 stage caused a precipitous, dose-dependent reduction in progeny viability in *smc-5* mutants (Figure 5a). Wild-type animals (N2) were remarkably resilient, with nearly all embryos hatching at both 0.15 mM and 0.4 mM MMS. By contrast, *smc-5(ok2421)* null mutants displayed sensitivity, with progeny viability plummeting to nearly zero at 0.4 mM. This hypersensitivity corroborates prior findings that the loss of the SMC-5/6 complex confers acute MMS sensitivity in *C. elegans*. The novel *smc-5* alleles also exhibited significant sensitivity to MMS, with viability sharply decreasing at 0.4 mM to levels approaching those of the null mutant. These results affirm that all *smc-5* mutants are severely compromised in repairing replication-associated DNA damage caused by MMS. *brc-1(tm1145)* worms, defective in intersister repair pathway, displayed slightly more tolerance to MMS than *smc-5(ok2421)*, [22] indicating *smc-5* null mutants were more sensitive to MMS than *brc-1* single mutants [22]. Conversely, the *mus-81(tm1937)* endonuclease mutant, deficient in the structure-specific Holliday junction resolvase MUS-81 [23,24], exhibited pronounced MMS sensitivity. The *lig-4(rb873)* mutant, defective in DNA ligase IV and non-homologous end joining (NHEJ), showed no significant MMS sensitivity, with progeny viability comparable to wild-type [22,25]. This lack of an MMS phenotype is expected, as NHEJ plays a minor role in repairing replication fork-associated double-strand breaks (DSBs) in *C. elegans* germ cells, where HR predominates.

Hydroxyurea (HU) treatment of L1 larvae provided complementary insights into replication stress responses in these strains (Figure 5b). Wild-type worms displayed robust tolerance to HU at the tested doses, whereas *smc-5* mutants exhibited moderate, dose-dependent sensitivity. These findings underscore the critical role of the SMC-5/6 complex in recovering from or repairing HU-stalled forks. Comparisons with repair-deficient controls under HU highlighted pathway-specific contributions. *brc-1(tm1145)* mutants exhibited no substantial progeny viability defects, consistent with the idea that HR is not a major mechanism for countering HU-induced damage, based on HU’s mode of action. By contrast, *mus-81(tm1937)* mutants showed moderate HU sensitivity. As with MMS, *lig-4(rb873)* mutants demonstrated no significant HU sensitivity, further confirming that NHEJ does not play a prominent role in repairing S-phase damage in *C. elegans* germ cells, which rely on high-fidelity HR. Collectively, the HU assays confirm that replication fork collapse necessitates SMC-5/6 function, with the novel *smc-5* alleles variably fulfilling this requirement. We next assessed progeny viability following L1 exposure to cisplatin, a genotoxic agent that introduces inter- and intra-strand crosslinks (ICLs). Cisplatin poses a stringent challenge to DNA repair pathways, as ICLs stall replication forks and require coordinated action by nucleotide excision repair, homologous recombination, and specialized nucleases for resolution. Consistent with its potency, cisplatin caused the most severe viability deficits among all treatments in our assay (Figure 5c). Wild-type animals managed to produce viable progeny at lower doses (50–100 µM) but exhibited complete lethality at higher concentrations. *smc-5(ok2421)* null mutants were exceptionally sensitive to cisplatin: at 50 µM, nearly all laid eggs failed to hatch, and at 100 µM and higher, *smc-5* mutants were essentially sterile. These results underscore the indispensable role of the SMC-5/6 complex in mitigating cisplatin-induced crosslinks [26].

The panel of repair-pathway mutants further elucidated ICL tolerance mechanisms. Similar to the *smc-5* mutants, the *mus-81(tm1937)* mutant displayed extreme sensitivity to cisplatin. This sensitivity reflects MUS-81’s critical role in generating recombinogenic DNA substrates necessary for downstream HR repair [23,24]. The *brc-1(tm1145)* mutant exhibited sensitivity comparable to N2 at 50 µM but showed zero viability at 100 µM, underscoring the essential role of HR under severe ICL damage [26]. The *lig-4(rb873)* NHEJ mutant displayed a similar sensitivity profile to *brc-1(tm1145)* under cisplatin treatment, reaffirming that NHEJ is not a primary pathway for ICL repair in *C. elegans* germ cells. For all these assays, a comparison between N2 and *nse-1::gfp(wsh1)* revealed no significant difference, indicating that the GFP tag did not influence the assay outcome (Figure S6). The L1-stage progeny viability assays strongly indicate that the SMC-5/6 complex is critical for surviving replication stress and DNA crosslink damage. The novel *smc-5* alleles provide invaluable tools for dissecting SMC-5/6 function. In summary, our results establish the SMC-5/6 complex as a multifaceted protector of genome integrity during replication. Its inactivation leads to dose-dependent defects in progeny viability under replication stress. The newly identified *smc-5* alleles variably impair these protective functions and illuminate the structure–function relationships within this essential complex.

### 3.7. Developmental Progression of C. elegans After L1 Exposure to Genotoxic Agents

**Figure 6:**
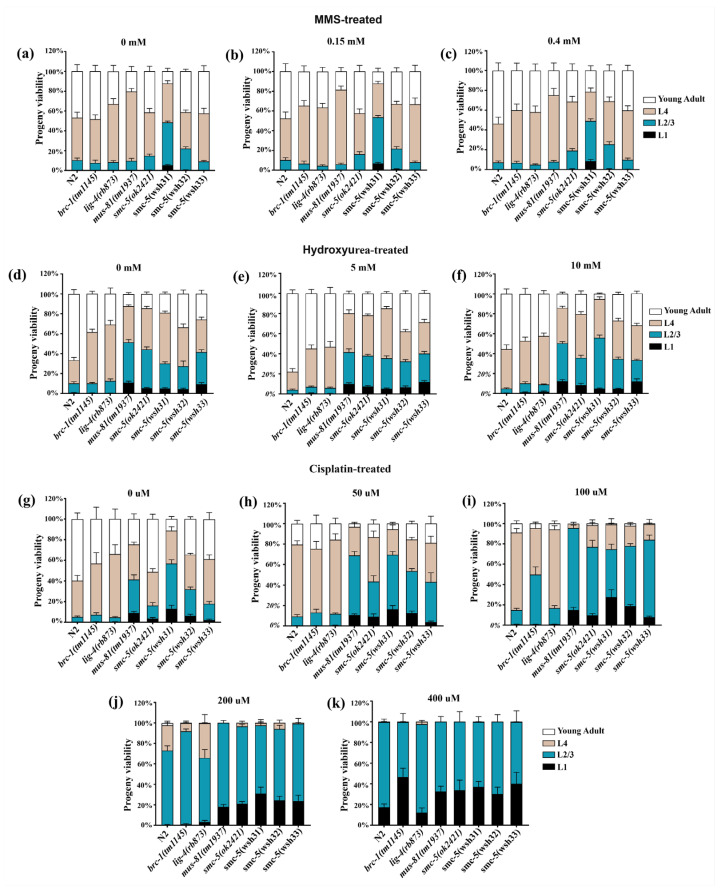
Developmental outcomes 48 h after L1-stage exposure to MMS, hydroxyurea, or cisplatin. Bars represent the percentages of animals reaching the indicated developmental stages at each treatment dose. Panels illustrate outcomes following exposure to (**a**–**c**) MMS (0, 0.15, 0.4 mM), (**d**–**f**) hydroxyurea (0, 5, 10 mM), and (**g**–**k**) cisplatin (0, 50, 100, 200, 400 µM). Sample sizes (n) are available in Tables S8–S10 for (**a**–**k**), respectively. No unusual difference was observed between N2 and *nse-1::gfp(wsh1)*, indicating that the GFP tag did not affect the assay outcome (Figure S7).

Testing developmental progression from the L1 stage, a period characterized by active cell division that makes worms particularly sensitive to DNA damage, is crucial. Such damage can delay growth to later stages and impair reproduction, consistent with earlier findings of reduced viability in Figure 5. To evaluate the impact of DNA damage on larval development, worms were synchronized at the L1 stage, exposed to graded doses of three genotoxic agents, and assessed for developmental progression after a 48 h recovery period. The proportion of animals reaching L2/L3, L4, or young adult stages (as well as those arrested at L1) was scored. Each panel in Figure 6 illustrates the developmental outcomes across genotypes. Overall, following 48 h of recovery from MMS treatment, virtually no observable differences between doses were detected (Figure 6a–c). However, at all doses, including controls without MMS treatment, only *smc-5(wsh31)* mutants exhibited a significant proportion of worms arrested at the L1 stage. The hydroxyurea (HU) group displayed a similar phenotype to that observed with MMS, with no apparent differences across doses (Figure 6d–f). Nevertheless, all *smc-5* mutants and *mus-81* exhibited L1 arrest at all doses. Except at the highest HU concentration (10 mM), where *brc-1(tm1145)* and *lig-4(rb873)* mutants showed few L1-arrested worms, they did not display L1 arrest at 15 and 10 mM HU.

Interestingly, worms demonstrated dose-dependent sensitivity to cisplatin (Figure 6g–j). At 400 µM, the cellular repair mechanisms of L1 worms appeared overwhelmed by cisplatin, as evidenced by prominent L1 arrest in the N2 group (Figure 6k). This concentration may not adequately represent cisplatin’s effects on development. The most informative result was observed at 200 µM, where exposure consistently led to the highest percentage of L1-arrested worms, with *smc-5(wsh31)* mutants displaying the highest. At this dose, N2 worms exhibited no L1 arrest, reflecting strong resilience. For all these assays, a comparison between N2 and *nse-1::gfp(wsh1)* revealed no significant difference, indicating that the GFP tag did not influence the assay outcome (Figure S7). These stage-specific observations emphasize the critical role of SMC-5/6 activity in enabling normal larval development after DNA damage. Furthermore, the new *smc-5* alleles provide a graded series of phenotypes, revealing variable effects on developmental recovery.

### 3.8. SMC-5 Y975D Ablates NSE-4 Binding and Impairs NSE-1 Chromosomal Localization

**Figure 7:**
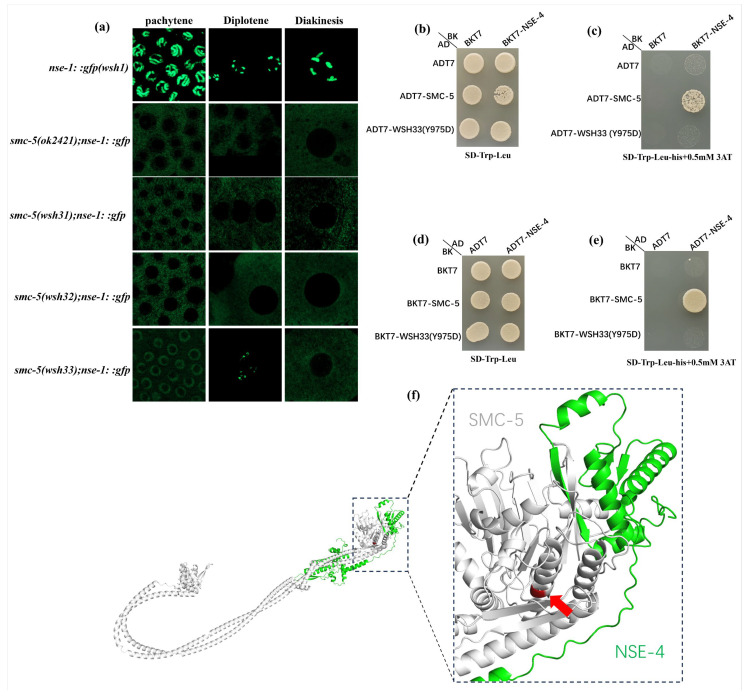
NSE-1::GFP localization depends on the SMC-5–NSE-4 interaction. (**a**) High-resolution fluorescence micrographs of *C. elegans* germline nuclei (pachytene, diplotene, and diakinesis stages) showing NSE-1::GFP localization in control and *smc-5* mutant backgrounds (scale bars, 10 μm). (**b**) Yeast two-hybrid (Y2H) control plate (SD –Trp –Leu) showing growth of all transformants, confirming they carry both SMC-5 and NSE-4 plasmids. (**c**) Y2H interaction test (SD –Trp –Leu –His + 3AT) with wild-type or Y975D mutant SMC-5 fused to the Gal4 activation domain (AD) and NSE-4 fused to the Gal4 DNA-binding domain (BD). (**d**) Y2H control plate for the reciprocal configuration (SD –Trp –Leu), where SMC-5 is in the BD vector and NSE-4 in the AD vector. (**e**) Selective interaction plate (SD –Trp –Leu –His + 3AT) for the reciprocal configuration. (**f**) The 3D model of the SMC-5/NSE-4 interface illustrating the position of the Y975D substitution.

To investigate how SMC-5 function influences NSE-1 localization in the germline, we analyzed high-resolution fluorescence images of NSE-1::GFP in *C. elegans* meiotic nuclei at various stages (pachytene, diplotene, and diakinesis; Figure 7a). In the wild-type control (*nse-1::gfp(wsh1)*), NSE-1::GFP was robustly associated with meiotic chromosomes throughout all stages. Pachytene nuclei displayed bright NSE-1::GFP signals uniformly localized on synapsed chromosomes, which persisted through diplotene and diakinesis. As chromosomes condensed into distinct bivalents during diakinesis, NSE-1::GFP remained concentrated on chromosomal structures, indicative of robust nuclear retention in the presence of functional SMC-5. In *smc-5* loss-of-function mutants, NSE-1::GFP failed to localize to chromosomes and was excluded from nuclei. Specifically, in the *smc-5(ok2421)* deletion and the truncation alleles *smc-5(wsh31)* (C587) and *smc-5(wsh32)* (Q655), NSE-1::GFP was no longer chromatin-enriched at pachytene, diplotene, or diakinesis. Instead, the signal appeared diffuse and predominantly extranuclear, with nuclear regions largely dark and faint GFP fluorescence evident in the cytoplasm. These observations align with prior reports that loss of SMC-5/6 complex components results in NSE-1 translocation out of the nucleus [10,11].

In contrast, the *smc-5(wsh33)* allele exhibited a partial, stage-dependent localization phenotype. At pachytene, NSE-1::GFP was delocalized from chromosomes but retained significantly more localization than in truncation mutants. Strikingly, at diplotene, NSE-1::GFP partially re-associated with chromosomes in *smc-5(wsh33)* mutants, suggesting that some NSE-1 was able to relocalize to chromatin despite the Y975D mutation. However, this rescue was incomplete and did not persist into later stages. By diakinesis, when chromosomes were fully condensed, NSE-1::GFP was absent from chromosomal structures in *smc-5(wsh33)* oocytes. Amino acid alignment revealed that tyrosine at position 975 (Y975) of SMC-5 is highly conserved (Figure 3f). In the *smc-5(wsh33)* mutant, this tyrosine is replaced by aspartic acid (D). Using yeast two-hybrid (Y2H) assays, we previously demonstrated that among the non-essential elements (NSEs) of the SMC-5/6 complex, only NSE-4 directly interacts with SMC-5 [27]. We hypothesized that the mislocalization of NSE-1::GFP observed in *smc-5(wsh33)* animals might be linked to a weakened SMC-5–NSE-4 interface.

To test this, we carried out yeast two-hybrid (Y2H) assays between SMC-5 and the kleisin subunit NSE-4 (Figure 7b–e). On non-selective medium (SD –Trp –Leu; Figure 7b) all transformants grew, confirming expression of both wild-type and Y975D SMC-5. On selective medium (SD –Trp –Leu –His + 3-AT) robust growth was seen for the wild-type SMC-5 + NSE-4 pair, whereas the Y975D combination failed to grow (Figure 7c). Reciprocal Y2H configurations produced the same pattern (Figure 7d,e). Because Tyr975 lies within the conserved C-terminal head domain of SMC-5 and points towards the domain core, its substitution is likely to perturb the overall head-domain fold. Such structural disruption could, in turn, impair several functions—including ATPase activity and interactions with other subunits such as NSE-2 and NSE-6—rather than selectively abolishing the contact with NSE-4. Our Y2H data therefore indicate that the Y975D mutation diminishes detectable SMC-5–NSE-4 binding in this assay, but they do not establish Tyr975 as a unique determinant of that interface. In summary, the Y975D substitution compromises SMC-5 function in Y2H assays, most plausibly by destabilizing the head domain and indirectly weakening multiple partner interactions, one of which is with NSE-4. Additionally, we generated a 3D model of the SMC-5/NSE-4 interface (Figure 7f). Red arrow points to the Y975D position in the SMC-5 protein. This model illustrates the position of the Y975D substitution, highlighting its proximity to the interface and, by implication, its potential to destabilize the SMC-5/NSE-4 interaction.

## 4. Discussion

In this study, we employed a forward genetic screen in *C. elegans* based on the localization of NSE-1::GFP to identify factors crucial for the function and chromosomal association of the SMC-5/6 complex. Our screen successfully isolated three independent mutants, *smc-5(wsh31)*, *smc-5(wsh32)*, and *smc-5(wsh33)*, all exhibiting aberrant NSE-1::GFP distribution in the germline. Subsequent genetic mapping and whole-genome sequencing pinpointed the causative lesions to the *smc-5* gene, identifying three novel alleles: *smc-5(wsh31)* (C587*), *smc-5(wsh32)* (Q655*), and *smc-5(wsh33)* (Y975D). These alleles provide a valuable resource for dissecting SMC-5 function, complementing the existing null allele *smc-5(ok2421)* [28]. A primary finding is the distinct impact of these *smc-5* alleles on the subcellular localization of NSE-1::GFP. The two truncating mutations, *smc-5(wsh31)* and *smc-5(wsh32)*, resulted in a phenotype closely resembling the *smc-5(ok2421)* null allele: NSE-1::GFP was excluded from the nucleus and failed to associate with meiotic chromosomes (Figure 3 and Figure 7a). This observation aligns with previous reports suggesting that the integrity of the core SMC-5/6 heterodimer is essential for the stable nuclear retention and chromatin loading of NSE subunits [6,15]. The premature stop codons in *wsh31* and *wsh32* likely lead to unstable or non-functional SMC-5 protein fragments incapable of proper complex assembly, resulting in the apparent cytoplasmic sequestration or degradation of NSE-1::GFP. Intriguingly, the missense allele *smc-5(wsh33)* (Y975D) conferred a distinct, partial, and stage-dependent NSE-1::GFP localization defect (Figure 3 and Figure 7a). While NSE-1::GFP was substantially delocalized from chromosomes at pachytene (though less severely than in truncation/null mutants), it exhibited partial re-association during diplotene, only to become mislocalized again by diakinesis. This dynamic and incomplete mislocalization suggests that the Y975D mutation compromises, but does not abolish, SMC-5 function related to NSE-1 recruitment or retention [18,19]. The Y975 residue is located within the C-terminal portion of the SMC domain, near the ATPase head region. Its striking conservation across diverse eukaryotic species (Figure 3f) underscores its functional importance [20,21]. The substitution of a neutral, aromatic tyrosine with a negatively charged aspartic acid (Y975D) likely disrupts critical interactions or local protein structure. This mutation likely perturbs the interaction between SMC-5 and the kleisin subunit NSE-4, which is known to bridge the SMC head domains and recruit NSE-1/3 [16,17,29]. A weakened SMC-5/NSE-4 interaction could destabilize the entire complex specifically on chromatin, leading to the observed partial and dynamic NSE-1 mislocalization. This missense allele thus provides a unique tool to probe the specific role of the SMC-5 C-terminal domain and the Y975 residue in complex stability and NSE-1 association.

Consistent with the essential nature of SMC-5/6, all three novel *smc-5* alleles caused significant defects in fundamental biological processes, including reduced brood size, decreased progeny viability, and slightly increased frequency of male progeny (indicative of X-chromosome nondisjunction) (Figure 3g–i). These phenotypes confirm that *wsh31*, *wsh32*, and *wsh33* represent substantial loss-of-function mutations impairing gametogenesis and chromosome segregation fidelity, core functions attributed to SMC-5/6 [30,31]. The slightly elevated male frequency seen in *smc-5(wsh31)*, *smc-5(wsh32)*, and *smc-5(wsh33)* but not in the null *smc-5(ok2421)* suggests that X-chromosome nondisjunction is triggered not by a simple loss of SMC-5, but by defective SMC-5 subunits that still enter the SMC-5/6 holocomplex. The nonsense (*wsh31*, *wsh32*) and missense (*wsh33*, Y975D) alleles retain the N-terminal arm yet lack, or disrupt, the C-terminal head that binds the kleisin NSE-4. The severity of the viability defect in the new alleles was comparable to the null mutant, suggesting that even the partial function potentially retained by *smc-5(wsh33)* is insufficient for normal embryonic development. It is also important to note that some differences observed in the *smc-5* mutant phenotypes might arise not solely from the absence of functional SMC-5 protein but also from potential toxicity associated with the different truncated or mutated SMC-5 proteins present in the system. The key differences among the alleles emerged from the DNA damage sensitivity assays. These assays revealed a spectrum of sensitivities, providing insights into the specific roles of SMC-5 domains in coping with different types of DNA lesions. At the L4 stage, *smc-5(wsh33)* displayed greater resilience to MMS compared to the truncation alleles *wsh31* and *wsh32*, which showed intermediate sensitivity. This suggests that the Y975D mutant protein retains partial function in repairing MMS-induced lesions. However, L1 stage assays, which probe replication-associated repair more directly, revealed profound sensitivity in all *smc-5* mutants, including *wsh33*, approaching the null phenotype at higher doses. This highlights the critical role of intact SMC-5/6 during replication for handling alkylation damage. The hypersensitivity of *smc-5* mutants compared to *brc-1(tm1145)* (HR deficient) aligns with models where SMC-5/6 acts downstream or in parallel to HR initiation to resolve repair intermediates [22]. The extreme sensitivity of *mus-81* mutants underscores the importance of structure-specific endonucleases in processing MMS-induced lesions, likely working in concert with SMC-5/6. The insensitivity of *lig-4* mutants confirms the minor role of NHEJ in this context in the germline.

The lack of significant HU sensitivity at the L4 stage is consistent with the fact that pachytene cells, the predominant stage assayed in L4s, are not actively replicating. Conversely, L1 stage exposure revealed moderate HU sensitivity in *smc-5* mutants (Figure 5b), directly implicating SMC-5/6 in managing stalled replication forks induced by nucleotide depletion [32,33]. Interestingly, *smc-5(wsh32)* (Q655*) appeared least sensitive among the *smc-5* alleles, suggesting potential specific roles for the N-terminal region versus the C-terminal domain affected in *wsh33*. The relative insensitivity of *brc-1* and *lig-4* mutants to HU, contrasted with the sensitivity of *mus-81* and *smc-5* mutants, suggests that resolving stalled/collapsed forks under HU stress primarily requires MUS-81 and SMC-5/6, rather than canonical HR initiation or NHEJ [23,24,25]. Cisplatin induced the most severe phenotypes. All *smc-5* alleles were hypersensitive, as viability at the L1 stage fell to nearly zero even at low doses (Figure 5c), and the effect was far stronger than in wild-type or in *brc-1* and *lig-4* mutants. This extreme sensitivity underscores that SMC-5/6 is indispensable not only for classical inter-strand cross-link (ICL) repair [34,35], but also for resolving DNA–protein cross-links (DPCs), lesions that likewise stall replication. In plants, a recent forward-genetic screen identified SMC6B as a key factor in DPC repair and showed that the SMC-5/6 complex promotes lesion removal through NSE2-dependent SUMOylation at the damage site, acting in parallel to the MUS81 nuclease and WSS1A protease pathways [36]. The pronounced cisplatin hypersensitivity of our *smc-5* mutants, including the null *smc-5(ok2421)* and truncation *smc-5(wsh32)*, is consistent with this conserved role. This is because cisplatin generates both ICLs and DPC-like adducts that demand coordinated nuclease, protease, and post-translational-modification activities for repair. The slightly milder response of the missense allele *smc-5(wsh33)* at the lowest doses suggests residual function, echoing partial-loss alleles in Arabidopsis that retain limited SUMO-ligation capacity. The high sensitivity of *mus-81* mutants in the same assay further supports the idea that SMC-5/6 operates in a pathway that intersects with, but is not redundant to MUS-81-mediated processing of cross-link-associated structures. Analysis of larval development after L1 exposure (Figure 6) reinforces these points: cisplatin caused a potent, dose-dependent arrest, and *smc-5* mutants were the most compromised. Notably, *smc-5(wsh31)* exhibited a persistent L1 arrest even without exogenous damage, implying that SMC-5/6 also mitigates endogenous DPC-like stress during development. Together with the Arabidopsis findings, our data highlight a conserved function for SMC-5/6 in safeguarding replication against both ICLs and DPCs, linking its SUMO-dependent activity directly to developmental progression following genotoxic insult [34,35].

Using yeast two-hybrid assays, we previously demonstrated that among the NSEs, only NSE-4 directly interacts with SMC-5. In wild type, NSE-1 and NSE-3 form a tight complex with the kleisin NSE-4, which together attach to the SMC-5/6 ATPase heads [29]. The Y975D mutation appears to specifically undermine this attachment. Yeast-2-hybrid experiments confirmed that SMC-5(Y975D) no longer binds NSE-4. Thus, Tyr975 of SMC-5 is essential for the SMC5–NSE4 interface that tethers the non-SMC subunits to the core SMC5/6 heterodimer. Our findings provide further validation of another study where yeast two-hybrid and structural analyses in *S. cerevisiae* have shown that the Nse4 kleisin bridges the head of Smc5 to the coiled-coil neck of Smc6 [29,37]. Structural and cryo-EM studies show that the kleisin subunit NSE4 uses its C-terminal domain to dock onto the bottom “cap” of the SMC5 head, while its N-terminal helix–turn-helix domain binds the head-proximal coiled-coil (“neck”) of SMC6 [9,37]. The Y975 residue lies in the region of SMC-5 where Nse4 is known to bind the Smc5 subunit [37]. This is supported by the 3D modeling of the SMC5–NSE4 interface (Figure 7f). Replacing this conserved tyrosine with an aspartate likely disrupts a critical contact or local hydrophobic environment needed for the Smc5–Nse4 interaction, thereby uncoupling the kleisin and associated NSE-1/NSE-3 subunits from the ring. Moreover, the interdependency of complex components is further highlighted by recent work showing that loss of *nse-1* leads to mislocalization of NSE-4 in *C. elegans* [10]. Our results present the converse scenario: a defective SMC-5 prevents proper localization of NSE-1 (and by implication NSE-4), underscoring that physical bridging of SMC-5 to NSE-4 is indispensable for holocomplex assembly on chromosomes. Given that mutations in various SMC-5/6 subunits are associated with genome instability disorders and cancer predisposition in humans, understanding the molecular pathology of the SMC-5(Y975D) complex can yield insight into how subtle perturbations in this complex can drive disease phenotypes. For example, our findings imply that therapeutic targeting of the Smc5–Nse4 interaction (or viral proteins that exploit this vulnerability) could severely compromise the complex’s ability to maintain genome integrity. Hence, the convergence of our genetic findings in *C. elegans* with human disease data underscores the critical, conserved role of the Smc5–kleisin interface for SMC5/6 complex function.

## 5. Conclusions

Our forward genetic screen successfully identified three novel *smc-5* alleles in *C. elegans* that disrupt NSE-1 localization and impair SMC-5/6 function. The characterization of these alleles, particularly the conserved Y975D missense mutation, provides significant insights into SMC-5 structure–function relationships. Our data demonstrate that the integrity of the SMC-5 protein, including specific residues like Y975, is critical not only for the stable association of NSE subunits with chromatin but also for organismal viability, chromosome segregation fidelity, and robust response to diverse genotoxic threats, especially those encountered during DNA replication. Future studies would focus on biochemically confirming the predicted effect of Y975D on SMC-5 interaction with NSE-4 and potentially other partners. Investigating the precise nature of the partial NSE-1::GFP re-localization in *smc-5(wsh33)* mutants during diplotene could reveal stage-specific regulatory mechanisms. Furthermore, epistasis analyses combining these *smc-5* alleles with mutations in different DNA repair pathways (HR, NHEJ, TLS, and NER) will help to precisely position SMC-5/6 within the complex network of genome maintenance. Examining chromosomal structures directly (e.g., using microscopy to look for bridges or fragments) in these mutants under stress conditions would provide further mechanistic insights. Overall, these alleles represent powerful tools for future investigations into the multifaceted roles of the essential SMC-5/6 complex in maintaining genome stability, a process fundamental to preventing developmental diseases and cancer.

## Data Availability

The original contributions presented in this study are included in the article/Supplementary Materials. Further inquiries can be directed to the corresponding authors.

## Supplementary Materials

The following supporting information can be downloaded at https://www.mdpi.com/article/10.3390/biom15060755/s1, Figure S1: Chromosome and interval mapping of mutations in *smc-5(wsh31)*; Figure S2: Chromosome and interval mapping of mutations in *smc-5(wsh32)*; Figure S3: Chromosome and interval mapping of mutations in *smc-5(wsh33)*; Figure S4: Amino acid residue changes in the new mutants; Figure S5: Sensitivities of N2 and *nse-1::gfp(wsh1)* to Genotoxic Agents; Figure S6: DNA damage sensitivity of L1-stage N2 and *nse-1::gfp(wsh1)*, measured by progeny viability; Figure S7: Developmental outcomes 48 h after L1-stage N2 and *nse-1::gfp(wsh1)* exposure to MMS, hydroxyurea, or cisplatin; Table S1: Brood size, progeny viability, and male percentage data; Table S2: MMS L4 assay sample size (n) for worms (and eggs); Table S3: HU L4 assay sample size (n) for worms (and eggs); Table S4: Cisplatin L4 assay sample size (n) for worms (and eggs); Table S5: MMS L1 assay sample size (n) for worms (and eggs); Table S6: HU L1 assay sample size (n) for worms (and eggs); Table S7: Cisplatin L1 assay sample size (n) for worms (and eggs); Table S8: MMS L1 developmental assay sample size for worms (n); Table S9: HU L1 developmental assay sample size for worms (n); Table S10: Cisplatin L1 developmental assay sample size for worms (n).
