# Supplementary material for: Separation-of-Function Alleles of smc-5 Reveal Domain-Specific Defects and a Conserved Residue Critical for Genome Maintenance
Source: Biomolecules. 2025 May 23;15(6):755. doi: 10.3390/biom15060755 (PMC12191127; doi:10.3390/biom15060755)
Supplement: Supplementary file 1 [file biomolecules-15-00755-s001.zip › biomolecules-3608657-supplementary-update.pdf]

Supplementary figures and tables

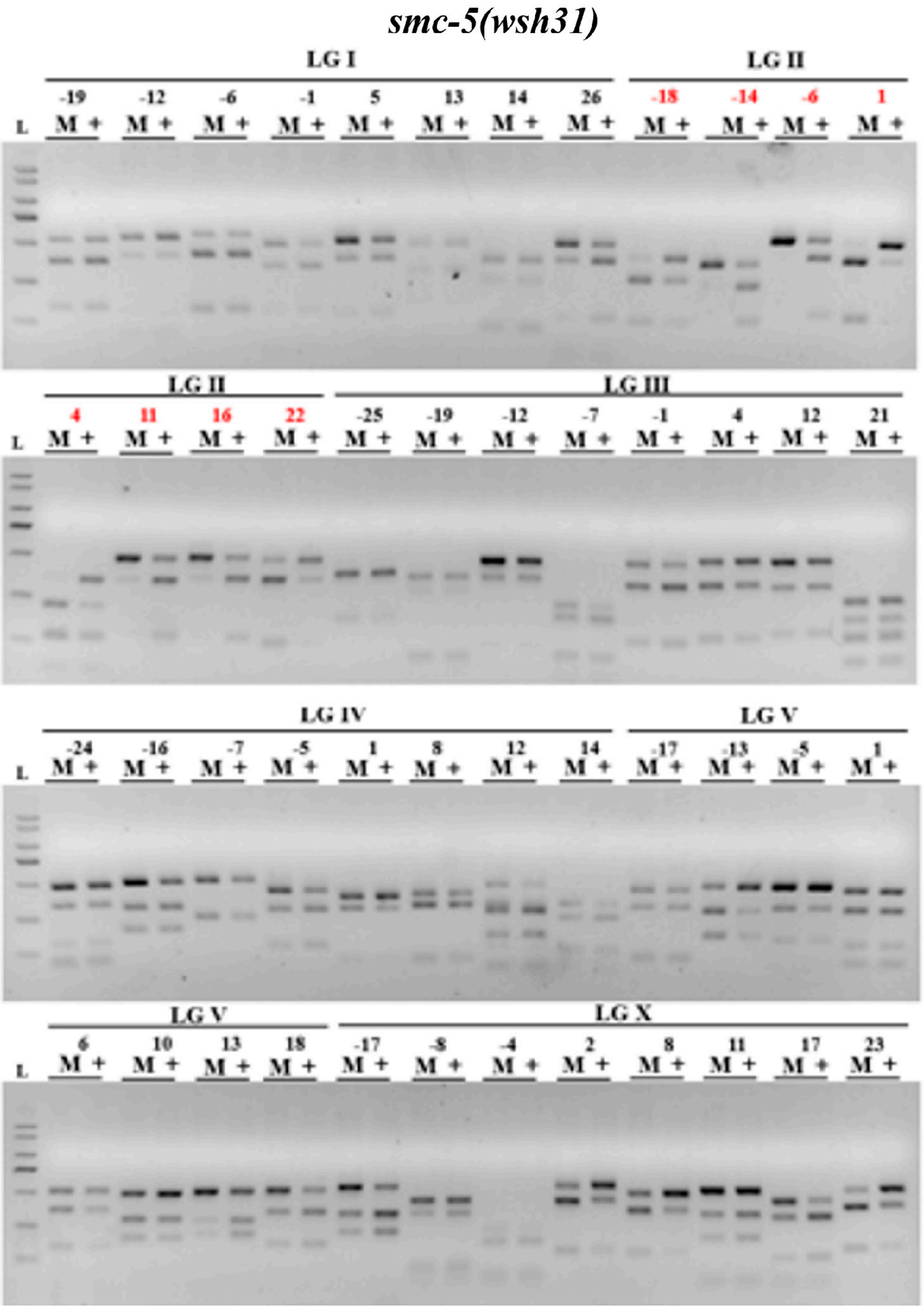

Figure S1. Chromosome and interval mapping of mutations in *smc-5(wsh31)*. Agarose gels display DraI-digested PCR products for 48 SNPs across chromosomes I, II, III, IV, V, and X. Lanes are labeled "M" (mutant) and "+" (wild-type).

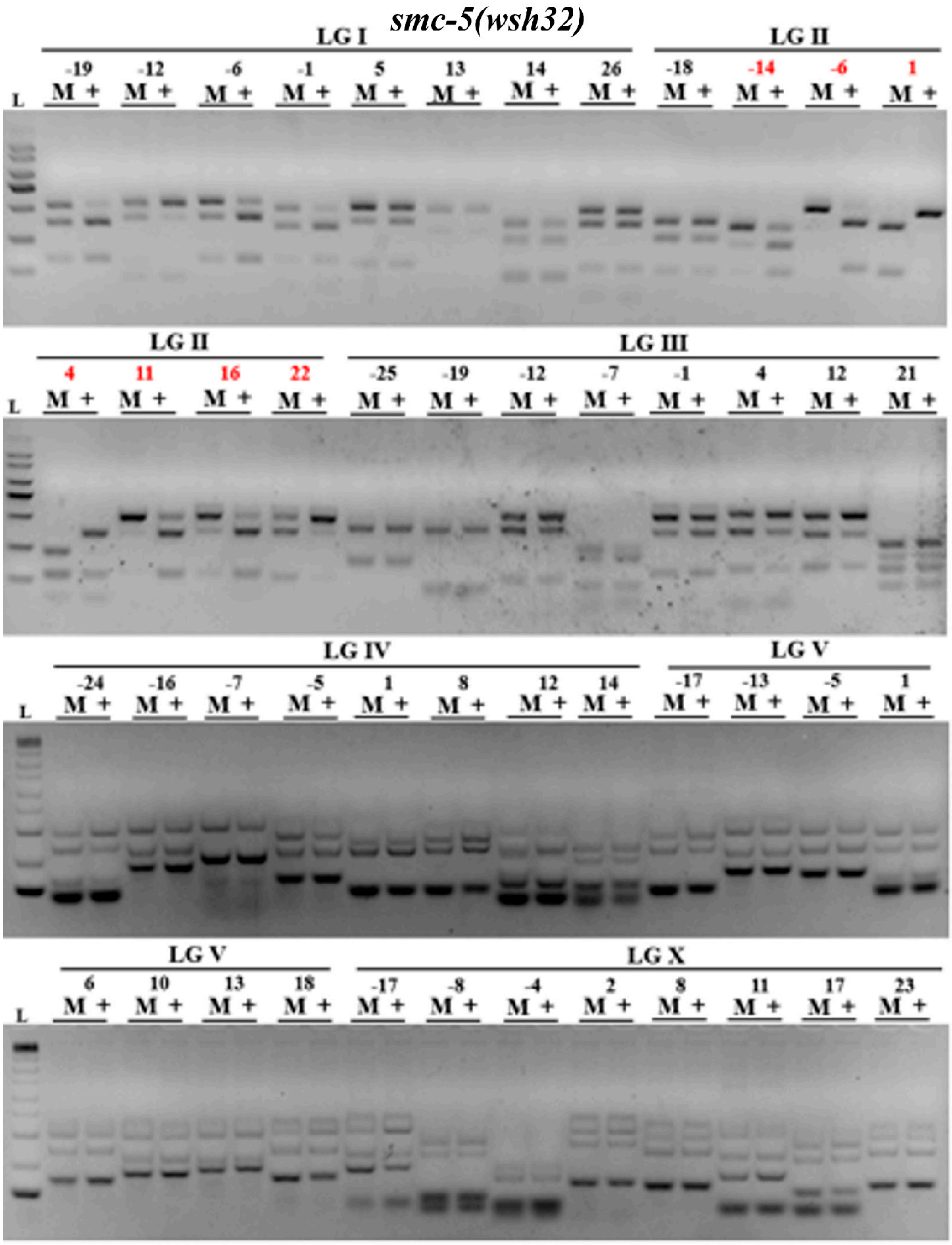

Figure S2. Chromosome and interval mapping of mutations in *smc-5(wsh32)*. Agarose gels display DraI-digested PCR products for 48 SNPs across chromosomes I, II, III, IV, V, and X. Lanes are labeled "M" (mutant) and "+" (wild-type).

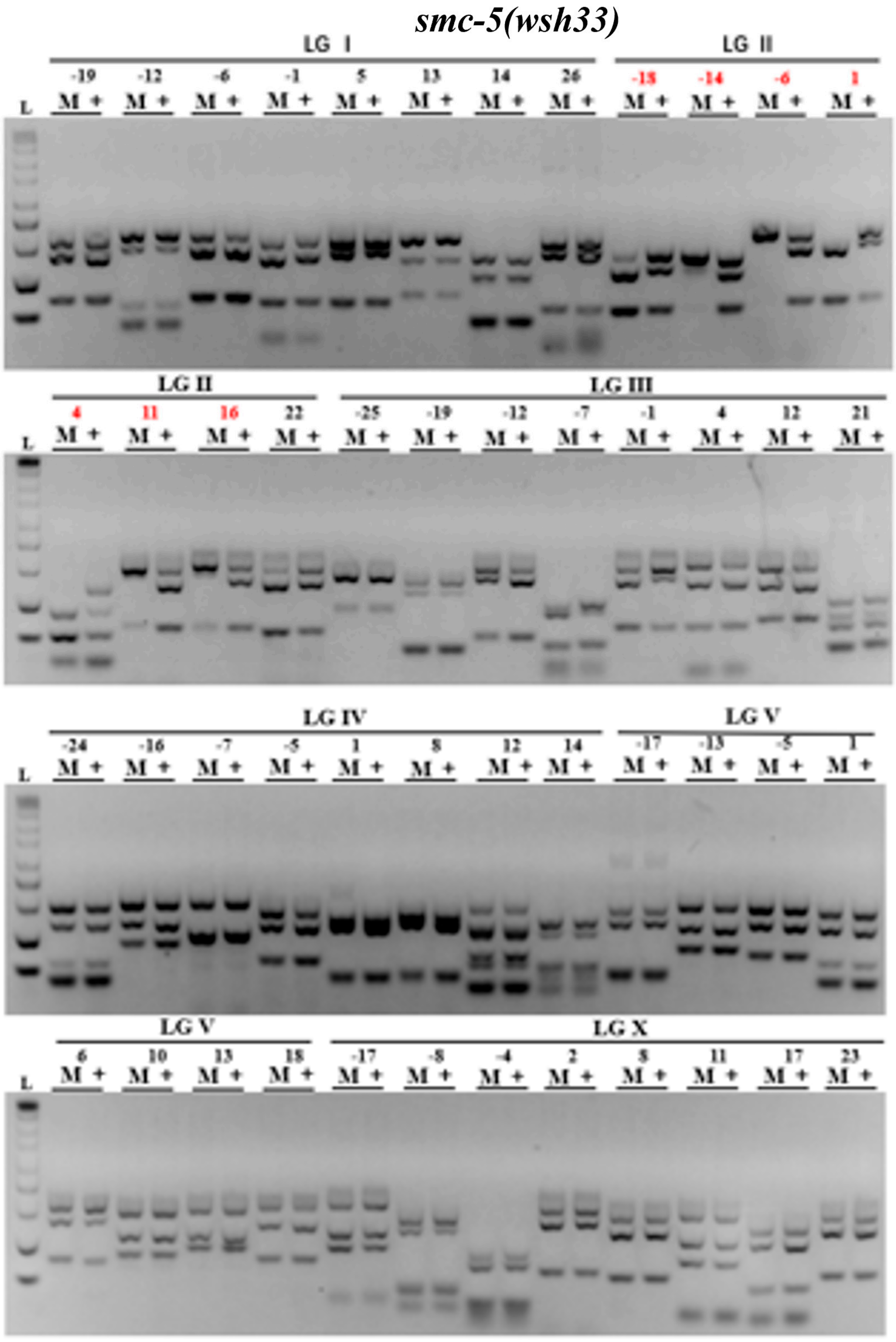

Figure S3. Chromosome and interval mapping of mutations in *smc-5(wsh33)*. Agarose gels display DraI-digested PCR products for 48 SNPs across chromosomes I, II, III, IV, V, and X. Lanes are labeled "M" (mutant) and "+" (wild-type).

(a)

MASQDSDEALPANYKDYPDGSLLRVVFHNFLTYEHTSFLPTASLNMILGHNGSGKSSIICGICLACGGSPKSLGRSERIVEYIRH
GCTEGYVEIAIADKQKGPQVVRLTIRVGEQPKYRLNDSATTQSEIADLRKHYNIQIDNPCAFLAQDKVKSFSEQSSIELLRNTEK
AASADLDQQHIDLMKQREDSTSIEDKCTTSENAIKRLEDEIGKIMPLVENYRKKLALQSKLRLLEKKMKIMEFEKFDREYKAELQ
NMDGAMIEYREVEKSIAECEKHRKNLEDRIKKDRSQISQMQRSCNEILAKVQEKGDKKLMEDMMQRAKAKLESAKKAADQ
HEKDVEKARKMIDQARARLQEAVDTLNGYEEFQSEMKSLEQKYSTAERDSRQEEDAIQKKSYEMRQLENKKRDEEQNSQLN
RQDRYRVLQNFSSDASKAYRWYQQNRSQFKGDVYMPIMDMVLKTPEAAKALENSVGVRDRTMFVCCYKEDELLINGKQH
SWRINTSVVPAEKIYSEDIDAQLPSELSRLGFKYLVSNCFDAPAPLKQFLCNVSGLNRIPFGGSDVEKKIAEVSQAIEQTRYSVFL
TANIRCQNSKSRYANNTLQSQSATREANTWRDQFFKVSPVAKRTDNSILEEIQKLKAEIDIRSEQLREKRGAIQKERDVLRQE
QMQWKSKKQVHTKWKTELASEMAKLEALENEVVDISAIEEEYANVEKKAILETKKMLENSIRWHKEIIDKHRLIGIFELSESICK
SRVNKSNSEAETHRSKLEDLKSVKDAAEDLLKTALNHKKAAASALMKECSLKTLDESKMSPAENKIYSSLVKMFEEADVPTD
MNTLDQAITSEKTRLKLAEDSGEDGSIVHEQRLKVLDDDLVLEKTRQEKLIENRARIHDKLGDEINNWRKEVETMIEQINVNY
VQFFDSLGCRGEVSLEVPENPLDIEKYGIMIMVCFRKGESMKRLDNKVQSGGERSVATMLYLLALQQLCPVPFRCIDEINQGM
DPTNERKVFDIMVGMWNGTTGTLSKTQYFLLSPKLLHGLDMRENVNIVMVNSTLTNSHGKHYDTSAKIDATFAKMGISA

C to Stop = *smc-5(wsh31)*

Q to Stop = *smc-5(wsh32)*

Y to D = *smc-5(wsh33)*

(b)

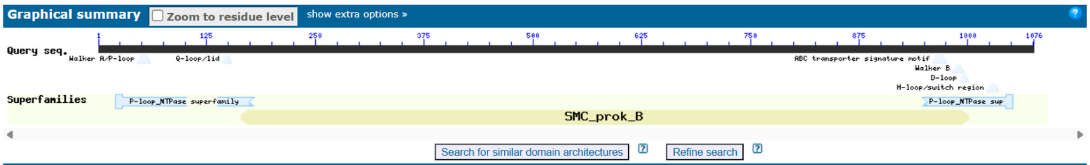

List of domain hits

Name	Accession	Description	Interval	E-value
[+] P-loop_NTPase super family	cl38936	P-loop containing Nucleoside Triphosphate Hydrolases; Members of the P-loop NTPase domain ...	947-1052	3.70e-54
[+] P-loop_NTPase super family	cl38936	P-loop containing Nucleoside Triphosphate Hydrolases; Members of the P-loop NTPase domain ...	20-180	1.35e-49
[+] SMC_prok_B super family	cl37069	chromosome segregation protein SMC, common bacterial type; SMC (structural maintenance of ...	165-1001	6.93e-22

Figure S4. (a) Amino acid residue changes in the new mutants. Sequence alignments of the *smc-5* gene in the wild-type (N2) and the three mutants, showing the specific sequence changes: C587* (TGT to TGA) in *smc-5(wsh31)*, Q655* (CAG to TAG) in *smc-5(wsh32)*, and Y975D (TAT to GAT) in *smc-5(wsh33)*. (b) Graphical map of SMC-5 structural domains.

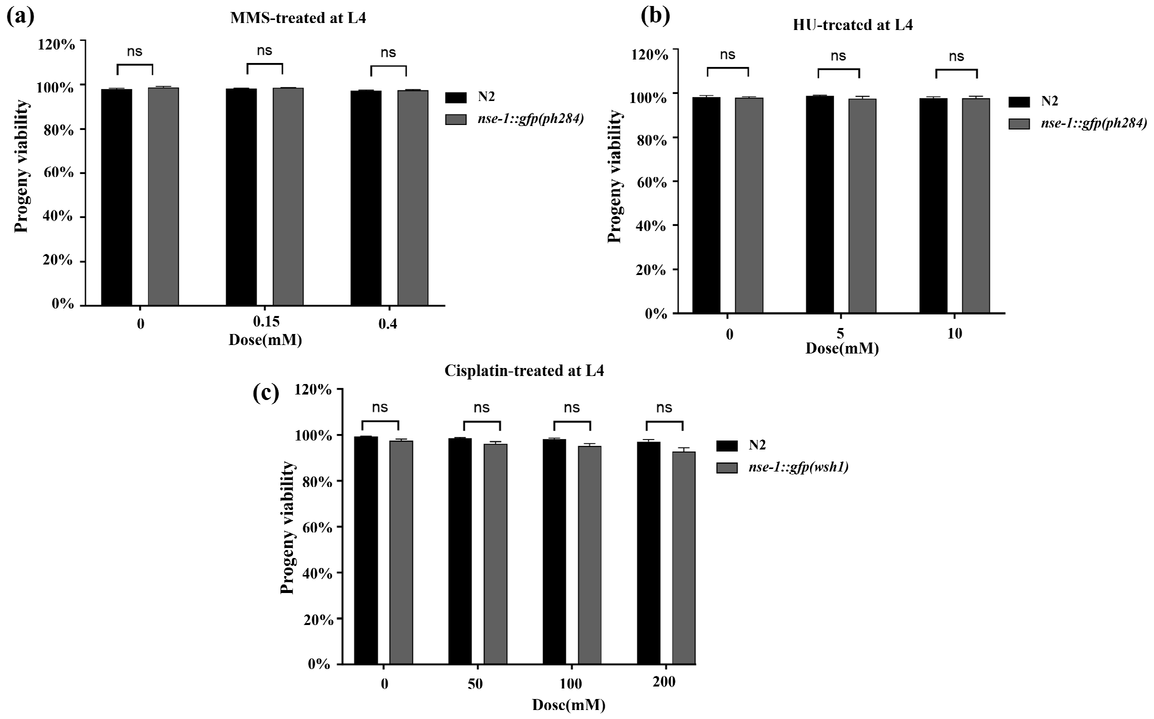

Figure S5. Sensitivities of N2 and *nse-1(wsh1)* to Genotoxic Agents (a) Viability assays of L4-stage N2 and *nse-1(wsh1)* exposed to varying concentrations of methyl methanesulfonate (MMS). (b) Viability assays of L4-stage N2 and *nse-1(wsh1)* subjected to replication stress induced by hydroxyurea (HU). (c) Viability assays of L4-stage N2 and *nse-1(wsh1)* exposed to escalating concentrations of cisplatin, which induces inter- and intra-strand DNA crosslinks. Bars represent the mean ± SEM for each genotype, based on three independent biological replicates. Statistical significance was evaluated using one-way ANOVA with multiple-comparison corrections. Asterisks indicate levels of significance relative to N2. ns: not significant). Sample sizes (n) are available at Table S2, Table S3, and Table S4 for (a), (b), and (c), respectively.

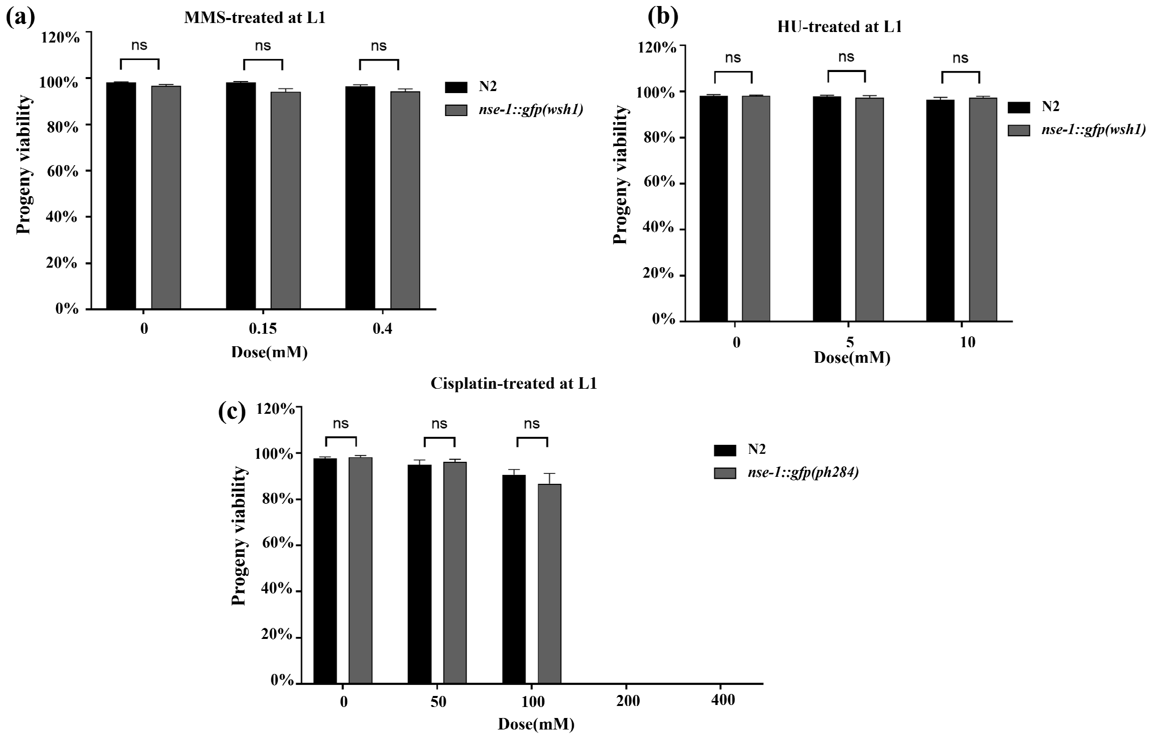

Figure S6. DNA damage sensitivity of L1-stage N2 and *nse-1(wsh1)*, measured by progeny viability Percent progeny viability following exposure to varying doses of (a) methyl methanesulfonate (MMS; alkylating agent), (b) hydroxyurea (HU; replication stress), and (c) cisplatin (inter- and intra-strand crosslinks). Sample sizes (n) are available at Table S5, Table S6, and Table S7 for (a), (b), and (c), respectively.

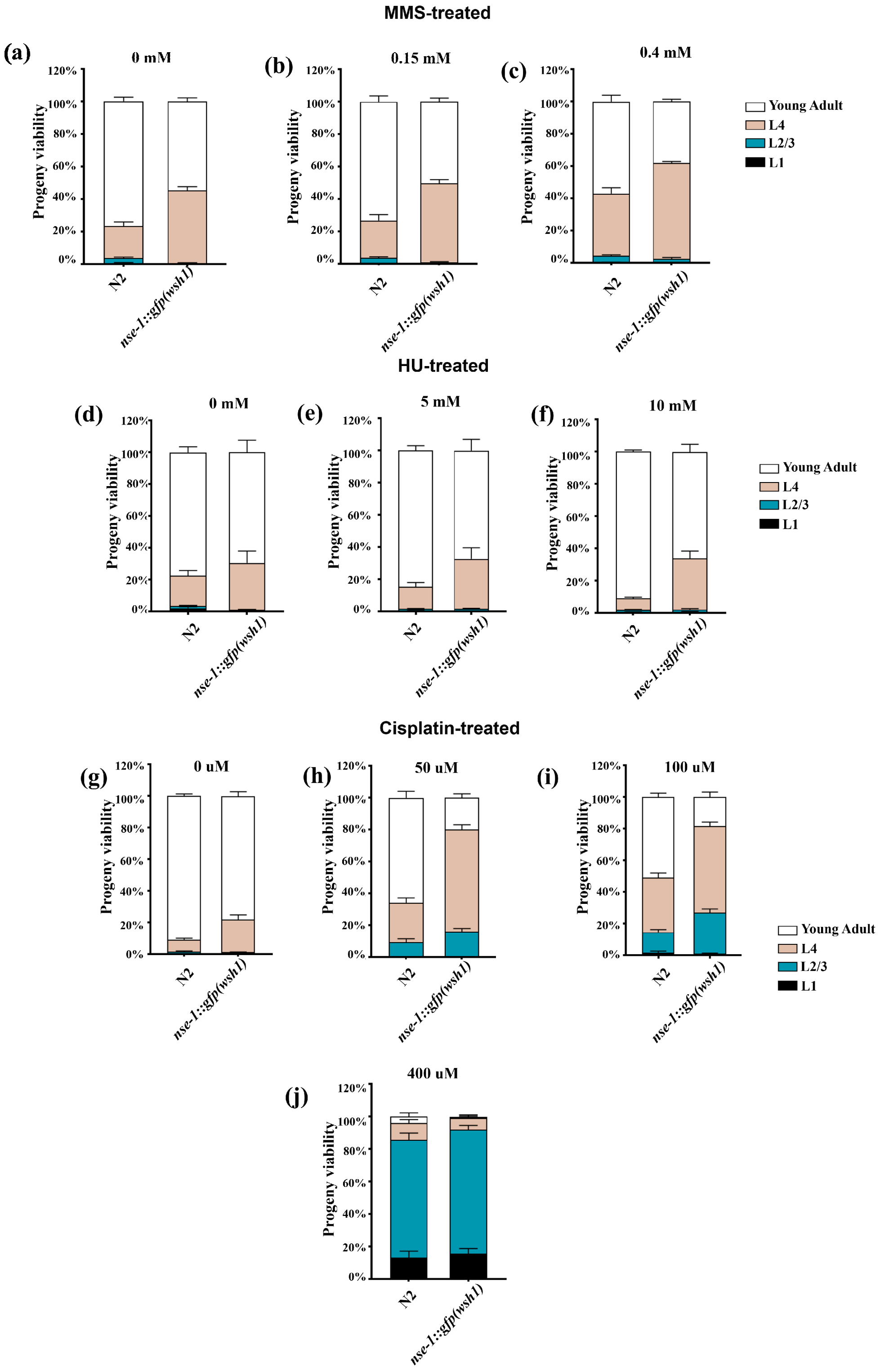

Figure S7. Developmental outcomes 48 hours after L1-stage N2 and *nse-1::gfp(wsh1)* exposure to MMS, hydroxyurea, or cisplatin. Bars represent the percentages of animals reaching the indicated developmental stages at each treatment dose. Panels illustrate outcomes following exposure to (a–c) MMS (0, 0.15, 0.4 mM), (d–f) hydroxyurea (0, 5, 10 mM), and (g–k) cisplatin (0, 50, 100, 200). Sample sizes (n) are available at Table S8, Table S9, and Table S10 for (a-c), (d-f), and (g-j), respectively.

Table S1. Brood size, progeny viability and male percentage data

	N2	*nse-1::gfp(wsh1)*	*smc-5(ok2421)*	*smc-5(wsh31)*	*smc-5(wsh32)*	*smc-5(wsh33)*
Total eggs laid	6991	4560	4784	2256	1896	1812
Total males	9	19	21	24	18	18
Average males	0.32±0.82	0.76±1.05	0.75±1.11	0.96±1.14	0.82±0.96	0.75±0.99
Average eggs laid（Brood size）	250±47.64	182±57.06	171±65.01	90±42.34	86±34.70	75±37.77
Average males/Average eggs (Average Male Percentage)	0.13%	0.42%	0.44%	1.06%	0.95%	0.99%
Progeny viability	96.91±2.08%	93.63±3.96%	74.16±10.89%	85.65±7.47%	64.96±20.56%	47.28±26.52%
N (sample size (n))	28	25	28	25	22	24

Table S2. MMS L4 assay sample size (n) for worms (and eggs)

strain	0 mM	0.15 mM	0.4 mM
N2	12（893）	12（844）	12（813）
mus-81(tm1937)	12（921）	12（759）	12（520）
smc-5(ok2421)	12（909）	12（997）	12（981）
smc-5(wsh31)	12（681）	12（646）	11（574）
smc-5(wsh32)	12（701）	12（610）	12（666）
smc-5(wsh33)	12（776）	12（695）	12（812）

N2 vs. nse-1::gfp(wsh1)

strain	0 mM	0.15 mM	0.4 mM
N2	12（1412）	12（1366）	12（1275）
nse-1::gfp(wsh1)	12（1478）	12（1479）	12（851）

Sample size: Number (n) of worms (and number of eggs (n))

Table S3. HU L4 assay sample size (n) for worms (and eggs)

strain	0 mM	5 mM	10 mM
N2	12（1084）	12（1142）	12（1117）
lig-4(rb873)	12（1078）	12（1074）	12（1099）
brc-1(tm1145)	12（852）	12（1076）	12（846）
mus-81(tm1937)	12（737）	12（781）	12（711）
smc-5(ok2421)	12（1013）	12（1224）	12（1238）
smc-5(wsh31)	12（751）	12（825）	12（834）
smc-5(wsh32)	12（789）	12（926）	12（973）
smc-5(wsh33)	12（913）	12（1020）	12（931）
N2 vs. nse-1::gfp(wsh1)			
strain	*0 mM*	*5 mM*	*10 mM*
N2	12（1184）	12（1183）	12（1157）
nse-1::gfp(wsh1)	12（1107）	12（1000）	12（886）

Sample size: Number (n) of worms (and number of eggs (n))

Table S4. Cisplatin L4 assay sample size (n) for worms (and eggs)

strain	0μM	50μM	100μM	200μM	400μM
N2	12（1180）	12（1240）	12（1003）	12（1244）	12（1109）
lig-4(rb873)	12（1080）	12（998）	12（946）	12（1053）	12（1005）
brc-1(tm1145)	12（971）	12（906）	12（777）	12（791）	12（835）
mus-81(tm1937)	12（1011）	12（1030）	12（807）	12（733）	12（805）
smc-5(ok2421)	12（1082）	12（1115）	12（1003）	12（892）	12（880）
smc-5(wsh31)	12（628）	12（609）	12（519）	12（724）	12（588）
smc-5(wsh32)	12（914）	12（1071）	12（893）	12（887）	12（886）
smc-5(wsh33)	12（1113）	12（1170）	12（887）	12（949）	12（884）
N2 vs. nse-1::gfp(wsh1)					
strain	0μM	50μM	100μM	200μM	400μM
N2	12（989）	12（978）	12（969）	12（939）	12（845）
nse-1::gfp(wsh1)	12（777）	12（662）	12（662）	12（701）	12（735）

Sample size: Number (n) of worms (and number of eggs (n))

Table S5. MMS L1 assay sample size (n) for worms (and eggs)

strain	0 mM	0.15 mM	0.4 mM
N2	12（939）	12（1006）	12（963）
lig-4(rb873)	12（861）	12（941）	12（923）
brc-1(tm1145)	12（718）	12（697）	12（624）
mus-81(tm1937)	12（647）	12（799）	12（692）
smc-5(ok2421)	11（474）	12（3）	12（0）
smc-5(wsh31)	12（707）	12（477）	12（328）
smc-5(wsh32)	12（510）	11（1）	12（0）
smc-5(wsh33)	12（840）	11（79）	12（6）

N2 vs. nse-1::gfp(wsh1)

strain	0 mM	0.15 mM	0.4 mM
N2	12（895）	12（904）	12（886）
nse-1::gfp(wsh1)	12（538）	12（400）	12（401）

Sample size: Number (n) of worms (and number of eggs (n))

Table S6. HU L1 assay sample size (n) for worms (and eggs)

strain	0 mM	5 mM	10 mM
N2	12（1898）	12（1647）	12（1370）
lig-4(rb873)	12（1580）	12（1579）	12（1270）
brc-1(tm1145)	12（1481）	11（1268）	12（1264）
mus-81(tm1937)	11（641）	11（754）	12（785）
smc-5(ok2421)	11（359）	12（202）	11（93）
smc-5(wsh31)	11（312）	11（209）	11（235）
smc-5(wsh32)	12（1139）	12（1010）	12（1021）
smc-5(wsh33)	12（285）	12（278）	12（356）

N2 vs. nse-1::gfp(wsh1)

strain	0 mM	5 mM	10 mM
N2	12（1338）	12（1167）	12（937）
nse-1::gfp(wsh1)	12（761）	12（600）	12（659）

Sample size: Number (n) of worms (and number of eggs (n))

Table S7. Cisplatin L1 assay sample size (n) for worms (and eggs)

strain	0μM	50μM	100μM	200μM	400μM
N2	12（971）	11（465）	12（67）	12（0）	12（0）
lig-4(rb873)	12（1055）	11（343）	12（0）	12（0）	12（0）
brc-1(tm1145)	12（834）	12（178）	12（0）	12（0）	12（0）
mus-81(tm1937)	12（448）	12（0）	12（0）	12（0）	12（0）
smc-5(ok2421)	12（273）	12（0）	12（0）	12（0）	12（0）
smc-5(wsh31)	12（283）	12（49）	12（0）	12（0）	12（0）
smc-5(wsh32)	11（155）	12（0）	12（0）	12（0）	12（0）
smc-5(wsh33)	11（323）	11（0）	11（0）	12（0）	12（0）
			N2 vs. nse-1::gfp(wsh1)		
strain	0μM	50μM	100μM	200μM	400μM
N2	12（975）	11（651）	12（393）	12（0）	12（0）
nse-1::gfp(wsh1)	12（323）	11（223）	12（160）	12（0）	12（0）

Sample size: Number (n) of worms (and number of eggs (n))

Table S8. MMS L1 developmental assay sample size for worms (n)

strain	0 mM	0.15 mM	0.4 mM
N2	1203	1433	1147
lig-4(rb873)	1310	1052	1521
brc-1(tm1145)	1020	865	1553
mus-81(tm1937)	1813	1298	2381
smc-5(ok2421)	979	1001	926
smc-5(wsh31)	1056	768	985
smc-5(wsh32)	867	920	918
smc-5(wsh33)	865	963	945
		N2 vs. nse-1::gfp(wsh1)	
strain	*0 mM*	*0.15 mM*	*0.4 mM*
N2	*1895*	*1581*	*1661*
nse-1::gfp(wsh1)	*1634*	*1453*	*1561*

Table S9. HU L1 developmental assay sample size for worms (n)

strain	0 mM	5 mM	10 mM
N2	1665	1631	1517
lig-4(rb873)	1531	987	1489
brc-1(tm1145)	1072	1000	1278
mus-81(tm1937)	1048	1072	1021
smc-5(ok2421)	1361	1481	1129
smc-5(wsh31)	878	792	774
smc-5(wsh32)	855	1138	1398
smc-5(wsh33)	661	581	753

N2 vs. nse-1::gfp(wsh1)

strain	0 mM	5 mM	10 mM
N2	1102	1273	1372
nse-1::gfp(wsh1)	875	1256	974

Table S10. Cisplatin L1 developmental assay sample size for worms (n)

strain	0μM	50μM	100μM	200μM	400μM
N2	1393	1483	1406	1466	1376
lig-4(rb873)	1907	1823	1599	1522	1034
brc-1(tm1145)	1145	1263	995	1004	835
mus-81(tm1937)	928	745	886	841	971
smc-5(ok2421)	1239	1332	1291	1145	1319
smc-5(wsh31)	848	672	701	839	833
smc-5(wsh32)	626	583	557	522	594
smc-5(wsh33)	1160	1271	1100	919	958

N2 vs. nse-1::gfp(wsh1)

strain	*0μM*	*50μM*	*100μM*	*200μM*	*400μM*
N2	*1795*	*1833*	*1728*	*1346*	*1157*
nse-1::gfp(wsh1)	*1589*	*1554*	*1370*	*1270*	*984*
